# Pathway enrichment and protein interaction network analysis for milk yield, fat yield and age at first calving in a Thai multibreed dairy population

**DOI:** 10.5713/ajas.18.0382

**Published:** 2018-07-26

**Authors:** Thawee Laodim, Mauricio A. Elzo, Skorn Koonawootrittriron, Thanathip Suwanasopee, Danai Jattawa

**Affiliations:** 1 Department of Animal Science, Kasetsart University, Bangkok 10900, Thailand; 2 Department of Animal Sciences, University of Florida, Gainesville, FL 32611-0910, USA

**Keywords:** Cattle, Gene Network, Multibreed, Single Nucleotide Polymorphism Marker, Tropical

## Abstract

**Objective:**

This research aimed to determine biological pathways and protein-protein interaction (PPI) networks for 305-d milk yield (MY), 305-d fat yield (FY), and age at first calving (AFC) in the Thai multibreed dairy population.

**Methods:**

Genotypic information contained 75,776 imputed and actual single nucleotide polymorphisms (SNP) from 2,661 animals. Single-step genomic best linear unbiased predictions were utilized to estimate SNP genetic variances for MY, FY, and AFC. Fixed effects included herd-year-season, breed regression and heterosis regression effects. Random effects were animal additive genetic and residual. Individual SNP explaining at least 0.001% of the genetic variance for each trait were used to identify nearby genes in the National Center for Biotechnology Information database. Pathway enrichment analysis was performed. The PPI of genes were identified and visualized of the PPI network.

**Results:**

Identified genes were involved in 16 enriched pathways related to MY, FY, and AFC. Most genes had two or more connections with other genes in the PPI network. Genes associated with MY, FY, and AFC based on the biological pathways and PPI were primarily involved in cellular processes. The percent of the genetic variance explained by genes in enriched pathways (303) was 2.63% for MY, 2.59% for FY, and 2.49% for AFC. Genes in the PPI network (265) explained 2.28% of the genetic variance for MY, 2.26% for FY, and 2.12% for AFC.

**Conclusion:**

These sets of SNP associated with genes in the set enriched pathways and the PPI network could be used as genomic selection targets in the Thai multibreed dairy population. This study should be continued both in this and other populations subject to a variety of environmental conditions because predicted SNP values will likely differ across populations subject to different environmental conditions and changes over time.

## INTRODUCTION

The Thai multibreed dairy population is primarily composed of crossbred animals with over 75% Holstein (91%) and the remainder comes from various *Bos indicus* (Red Sindhi, Sahiwal, Brahman, and Thai Native) and *Bos taurus* (Brown Swiss, Red Danish, and Jersey) breeds [1]. Recent genome-wide association studies (GWAS) in Thailand found sets of significant single nucleotide polymorphism (SNP) markers from GeneSeek 9K chip associated with genes affecting lactation characteristics, milk yield (MY), fat yield (FY), and age at first calving (AFC) that were mostly different from those found in *Bos taurus* breeds in temperate regions [2,3]. Use of low-density in the Thai studies (9K) and high density in the studies in temperate regions (50K to 770K) may have been largely responsible for these differences. Unfortunately, budgetary restrictions have allowed only a small fraction of the animals in the Thai multibreed dairy population to be genotyped with GeneSeek 80K. An efficient alternative to increase the numbers of SNP per animal without increasing the cost of genotyping SNP is genomic imputation. Jattawa et al [4] found that program FImpute was more accurate than Findhap and Beagle software when imputing from GeneSeek 9K, 20K, and 26K to 80K in the Thai multibreed dairy population. Thus, imputation with FImpute of all genotyped animals with low-density chips could help increase the accuracy of estimation of SNP marker effects and the likelihood of identifying SNP markers associated with genes affecting dairy traits in this population. Further, because only a fraction of animals with phenotypes have genotypes, computation of SNP marker effects and explained genomic variation could be accomplished by utilizing the single-step genomic best linear unbiased prediction (ssGBLUP) developed at the University of Georgia [5].

The GWAS for milk production and reproductive traits in Holstein in temperate regions identified regions associated with MY, FY, and AFC in all autosomes [6–8]. Similarly, GWAS in Thailand found a largely different set of significant SNP distributed across all 29 autosomes and the X chromosome associated with milk production and reproductive traits in the Holstein upgraded Thai multibreed dairy population [2,3]. However, GWAS provide limited information on relationships among genes affecting quantitative traits. Analysis of gene networks and biological pathways would provide a more comprehensive understanding of the sets of genes affecting multiple milk production and reproduction traits in dairy cattle. Biological pathway research in Holstein indicated that most sets of genes associated with milk production in these studies were involved in metabolic pathways, fat digestion and absorption, arginine and proline metabolism and tight junctions [7]. However, sets of genes involved in biological pathways related to milk production may be influenced by population structure and selection [8]. As with differences in sets of SNP associated with milk production and reproduction traits between the multibreed cattle in Thailand and Holstein cattle in temperate zones [2,3], biological pathways and gene networks associated with these traits may also differ in Thai multibreed and purebred Holstein dairy populations. Thus, the objectives of this study were to determine biological pathways and protein-protein interaction (PPI) gene networks associated with MY, FY, and AFC in the Thai multibreed dairy population under tropical environmental conditions.

## MATERIALS AND METHODS

### Animals, management and traits

This research utilized 8,361 first-lactation cows from 810 farms located in the Northern, Northeastern, Central, Western, and Southern regions of Thailand. These cows were the progeny of 1,210 sires and 6,992 dams. Eighty-eight percent of animals in the database were Holstein (H) crossbreds (75% H and above); the remaining 25% belonged to other breeds (O) including Jersey, Brown Swiss, Red Danish, Sahiwal, Red Sindhi, Brahman, and Thai Native.

Cows were housed in open barns where they had access to roughage, concentrate and a mineral supplement. Green roughage consisted of freshly cut grasses (cut and carry) including Napier grass (*Pennisetum purpureum*), Guinea grass (*Panicum maximum*), Ruzi grass (*Brachiaria ruziziensis*), and Para grass (*Brachiaria mutica*). Cows were fed approximately 30 to 40 kg/d of roughage and 5 to 10 kg/d of concentrate, or equivalently, 1 kg of concentrate per 2 kg/milk produced. The concentrate (14% to 22% of crude protein and 63% to 83% of nitrogen-free extract) was provided twice per day during milking (morning: 4:30 to 7:00 am and afternoon: 2:30 to 4:30 pm). Agricultural byproducts (rice straw, pineapple waste and sweet corn cob or husk), hay, and (or) silage were used as supplements as green roughage decreased in winter and summer [1].

Traits in this research were 305-d MY, 305-d FY, and AFC. Test-day MY and fat percentage were collected monthly from individual first-lactation cows between 1989 and 2014. Test-day FY was computed as the product of fat percentage and MY. Subsequently, monthly test-day MY and FY were used to compute MY and FY using the test-interval procedure [9,10].

### Genomic DNA and genotypic data

Blood and semen samples were collected from 2,661 animals (89 sires and 2,572 dams) of the Thai multibreed dairy population. Genomic DNA was extracted from blood using a MasterPure DNA Purification kit for blood version II (EPICENTRE Biotechnologies, Madison, WI, USA) and from semen using a GenElute Mammalian Genomic DNA Miniprep Kit (Sigma, Ronkonkoma, NY, USA). The DNA quality was assessed with a NanoDrop 2000 spectrophotometer (Thermo Fisher Scientific Inc., Wilmington, DE, USA). DNA samples from all animals (n = 2,661) were ensured to contain sufficient DNA for genotyping (absorbance ratio of approximately 1.8 at 260/280 nm and DNA concentration higher than 15 ng/μL). DNA samples were genotyped with GeneSeek Genomic Profiler (GGP) 9K, 20K, 26K, and 80K chips (GeneSeek Inc., Lincoln, NE, USA).

Animals genotyped with GGP9K, GGP20K, and GGP26K were imputed to GGP80K using program FImpute version 2.2 [4,11]. The imputed markers were subjected to quality control prior to further analysis. Quality control consisted of removing imputed markers with call rates lower than 90% and minor allele frequencies lower than 0.01. The resulting edited file contained 75,776 SNP markers per genotyped animal.

### Genome-wide association analysis

A GWAS for MY, FY, and AFC was performed using ssGBLUP [5]. Animals with phenotypes and genotypes as well as animals with only phenotypes were included in this analysis. A 3-trait genomic-polygenic model was used to obtain genetic variances for and covariances between MY, FY, and AFC. Fixed effects included contemporary group (herd-year-season), breed regression effect (as a linear function of expected O fraction in each animal, where O = other breeds, including Brown Swiss, Red Danish, Jersey, Red Sindhi, Sahiwal, Brahman, and Thai Native), and heterosis regression effect as a linear function of heterozygosity (expected H fraction in the sire times expected O fraction in the dam plus expected O fraction in the sire times expected H fraction in the dam). Random effects were animal additive genetic and residual. The mean for random animal additive genetic and residual effects was assumed to be zero. The variance-covariance matrix among animal additive genetic effects for MY, FY, and AFC was equal to H⨂V_a_ where H was the genomic-polygenic relationship matrix, V_a_ was variance-covariance matrix among additive genetic effects for these traits, and ⨂ was the Kronecker product. Residual variance-covariance matrix was equal to I⨂V_e_ where I was identity matrix and V_e_ was variance-covariance matrix among residual effects. The H equal to $\left[\begin{array}{cc} {\text{A}}_{11}+{\text{A}}_{12}{\text{A}}_{22}^{-1}({\text{G}}_{22}-{\text{A}}_{22}){\text{A}}_{22}^{-1}{\text{G}}_{21} & {\text{A}}_{12}{\text{A}}_{22}^{-1}{\text{G}}_{22}\\ {\text{G}}_{22}{\text{A}}_{22}^{-1}{\text{A}}_{21} & {\text{G}}_{22} \end{array}\right]$, where A_11_ was the submatrix of additive relationships between non-genotyped animals, A_12_ was the submatrix of additive relationships between genotyped animals, ${\text{A}}_{22}^{-1}$ was the inverse of the matrix of additive genetic relationships between genotyped animals, and G_22_ was the matrix of genomic relationships among genotyped animals [12]. Matrix G_22_ = ZZ′/2 ∑ p_j_ (1 – p_j_), where p_j_ was the frequency of the second allele in locus j and Z was the incidence matrix of SNP effects whose elements were defined as z_ij_ = (0 – 2p_j_) if the genotype for locus j was homozygous 11, z_ij_ = (1 – 2p_j_) if the genotype for locus j was heterozygous 12 or 21 and z_ij_ = (2 – 2p_j_) if the genotype for locus j was homozygous 22. Matrix G_22_ was scaled using the default parameters of the BLUPF90 Family of Programs [13], i.e., the default scaling of matrix required the mean of the diagonal elements of G_22_ to be equal to the mean of the diagonal elements of A_22_ and the mean of the off-diagonal elements of G_22_ to be equal to the mean of the off-diagonal elements of A_22_.

Variance and covariance components for MY, FY, and AFC were estimated using restricted maximum likelihood procedures and computed via program AIREMLF90 [14] using an average information algorithm. Program POSTGSF90 was used to calculate the proportion of genetic variance explained by each SNP, additive SNP marker effect and construct Manhattan plots of percentages of the genetic variance explained by individual SNP. The percentage of the genetic variance explained by each SNP was calculated as the ratio of the variance explained by that SNP divided by the total genetic variance [15]. The predicted value of SNP associated with genes was calculated as sum of the additive SNP markers effect for each gene.

### Identification of genes associated with milk yield, fat yield, and age at first calving

Individual SNP that explained at least 0.001% of the genetic variance for MY, FY, and AFC were selected to determine potential genes associated with these traits. The position of these SNP markers in base pairs was used to locate genes or nearby genes in the UMD *Bos taurus* 3.1 assembly of the bovine genome at the National Center for Biotechnology Information (NCBI) using R package Map2NCBI [16]. Only SNP inside or within 2,500 bp of genes in the NCBI database were utilized for the pathway enrichment and (PPI) network analyses.

### Pathway enrichment analysis

Genes associated with MY, FY, and AFC were used to identify biological pathways in *Bos taurus* at the Kyoto encyclopedia of genes and genomes database using the ClueGo plugin of Cytoscape [17]. The statistical test used for the pathway enrichment analysis by ClueGo was a right-sided test based on the hypergeometric distribution corrected for multiple testing with the Bonferroni step-down method. Significantly enriched pathways for these traits were defined to be those with p<0.05.

### Protein-protein interaction network analysis

The name of genes for MY, FY, and AFC was used to identify PPI from neighborhood, co-occurrence, gene fusions, co-expression, experiments, databases, and text mining using program STRING [18]. The STRING defined PPI as a probabilistic confidence score. A high confidence score implied that interactions between proteins from the database could be considered as valid edges in a network. Thus, only PPI with a high confidence score (>0.7) were used to construct the PPI network. The PPI network was visualized using Cytoscape [19]. The CytoNCA plugin for Cytoscape was used to analyze the number connections between genes in the PPI network [20].

## RESULTS AND DISCUSSION

### Genetic variance explained by individual single nucleotide polymorphism and chromosomes

**Figure 1:**
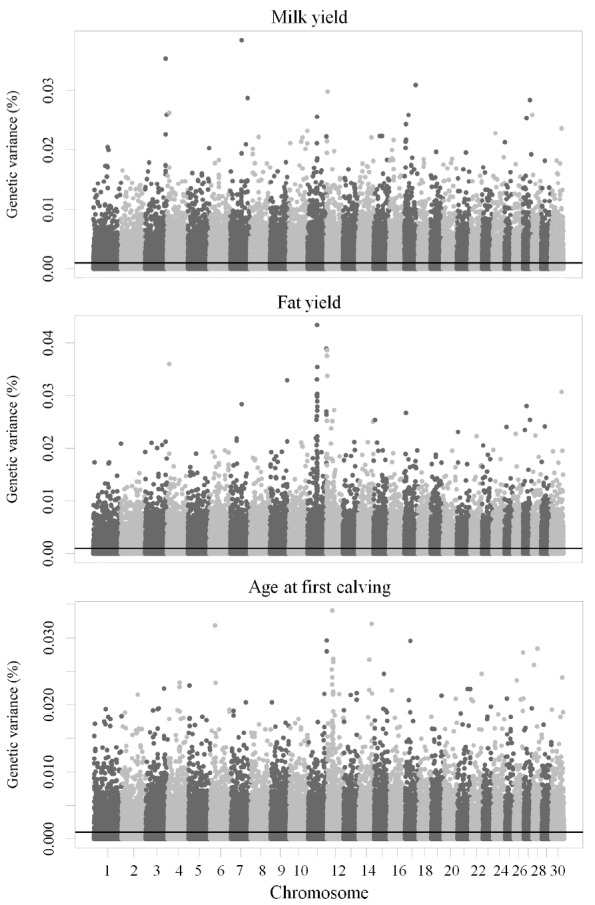
Manhattan plot of the percent of the genetic variance for milk yield, fat yield, and age at first calving explained by each SNP. The black line is the threshold for SNP accounting for more than 0.001% of the genetic variance for these traits. SNP, single nucleotide polymorphism.

The percentage of genetic variance explained by each SNP are shown in Figure 1. Most SNP markers (65%) explained less than 0.001% of the genetic variance each and together they accounted for 13% of the genetic variance. Conversely, 35% of SNP markers that explained 0.001% or more of the genetic variance and accounted for the largest fraction (87%) of the total genetic variance for MY, FY, and AFC. SNP markers were located inside genes, within 2,500 bp, between 2,500 and 5,000 bp, between 5,000 and 25,000 bp and beyond 25,000 bp of genes in the NCBI database (Supplementary Table S1). The percent of SNP inside genes or within 2,500 bp of genes explaining at least 0.001% of the genetic variance was 44% for MY, and FY, 43% for AFC, and accounted for 38% of the genetic variance for these traits.

**Figure 2:**
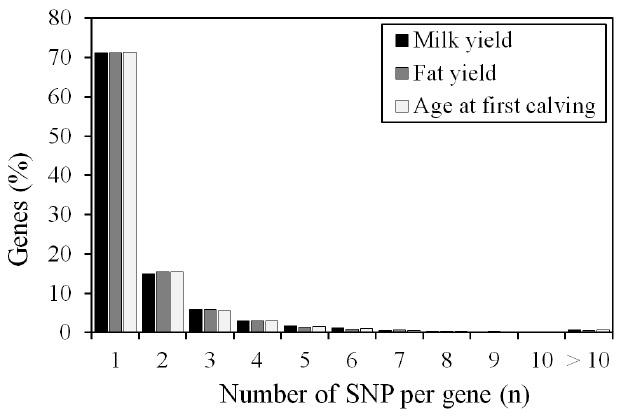
Distribution of genes associated with milk yield, fat yield and age at first calving by number of SNP per gene. SNP, single nucleotide polymorphism.

Numbers of SNP per gene ranged from 1 to 37 for MY, 1 to 25 for FY, and 1 to 29 for AFC (Figure 2). Seventy one percent of SNP associated with these traits had a one to one correspondence with genes in the NCBI database indicating that the vast majority of SNP markers in this population pointed to a single gene within the genome.

Numbers of genes and total genetic variance per chromo some for MY, FY, and AFC identified by SNP genotypes inside or within 2,500 bp of genes in the NCBI database are shown in Supplementary Table S2. The genetic variance explained by each chromosome ranged from 0.66% (chromosome 27) to 2.02% (chromosome 5) for MY, 0.58% (chromosome 27) to 2.09% (chromosome 11) for FY, and 0.58% (chromosome 27) to 2.01% (chromosome 4) for AFC. These low percentages of explained genetic variance indicated that MY, FY, and AFC were influenced by large numbers of genes accounting for small amounts of genetic variation scattered throughout the genome.

**Figure 3:**
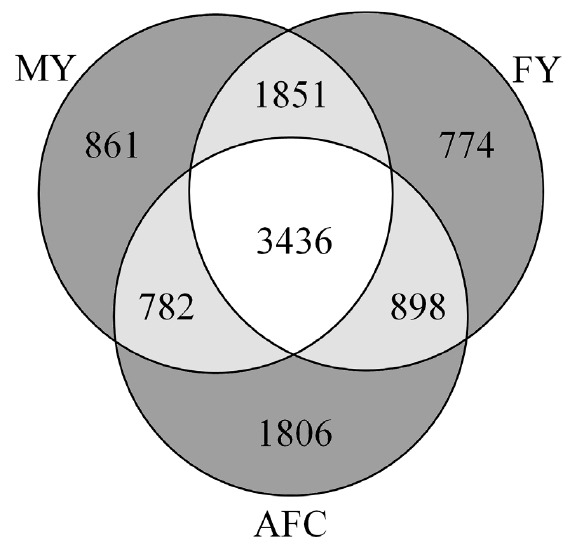
Number genes associated with one (dark gray), two (light gray) and three (white) traits in the Thai multibreed population. MY, milk yield; FY, fat yield; AFC, age at first calving.

Figure 3 shows numbers of genes associated with only one trait (dark gray), two traits (bright gray), and all three traits (white) based on Map2NCBI allocations. Numbers of single-trait gene associations (861 for MY, 774 for FY and, 1806 for AFC) were lower than two-trait gene associations (1,851 for MY and FY, 782 for MY and AFC, and 898 for FY and AFC) and three-trait gene associations (3,436 for MY, FY, and AFC). This indicated that genes were likely to be involved in multiple-trait associations than single-trait associations. These associations offer a biological rationale for the existence of genetic correlations among these traits. All genes associated with all three traits were located in the 29 autosomes and the X chromosome. The percentage of the genetic variance explained by these genes across all chromosomes was 26.2% for MY, 26.3% for FY, and 24.7% for AFC (Supplementary Table S3). These results provide additional evidence for these three quantitative traits (MY, FY, and AFC) to be determined by sets of genes spread across the genome in the Thai multibreed [2,3] and Holstein populations [7].

### Pathway enrichment analysis

Enriched pathways were classified into four categories: cellular processes, nervous system, digestive system, and environment adaptation are shown in Table 1. The genetic variance explained by the genes involved in these 16 significantly enriched pathways was 2.63% for MY, 2.59% for FY, and 2.49% for AFC (Table 1). The total predicted value of the SNP associated with these genes (as deviations from the second allele at each locus) were second allele at each locus had a larger effect than the first allele at most loci for MY and FY, and that the opposite occurred for AFC.

#### Cellular processes

Cellular process pathways related to cell proliferation, differentiation, migration, survival and apoptosis are essential for physiological changes in the ovarian follicle and mammary gland [21]. Therefore, cellular processes pathways contained the largest number of genes (266) and accounted for the largest percentage of the genetic variance for MY (2.24%), FY (2.29%), and AFC (2.12%) of all categories of significantly enriched pathways (Table 1). The sum of the predicted values of the SNP associated genes in enriched cellular pathways were −3.7513 for MY, −0.1524 for MY, and 0.0128 for AFC (Table 2), indicating that allele 2 at each locus had a larger effect than allele 1 for MY and FY, but a smaller effect than allele 1 for AFC.

Ovarian follicle and mammary gland development are influenced by the calcium-signaling pathway, which in turn is regulated by growth factors through changes in the concentration of free calcium ions (Ca^2+^). Specifically, Ca^2+^ acts as an activator in the mitogen-activated protein kinase (MAPK) signaling pathway in ovarian follicle and mammary gland cells [22]. The MAPK links extracellular signals to the machinery that controls many fundamental cellular processes such as cell inflammation, proliferation, metabolism, motility, and apoptosis [23]. Extracellular signal-regulated kinase 5, a member of the MAPK family, mediates the production of prolactin [24], a regulator in the development of the mammary gland. The MAPK signaling pathway was found to be essential for the development of ovarian follicles in heifers [21] and the mammary gland during lactation in Holstein [6,24] and Jersey [6]. The MAPK pathway is regulated by proteins from three associated pathways: Ras-related protein 1 from the Rap1 signaling pathway (http://www.genome.jp/dbgetbin/www_bget?pathway:bta04015), Ras proteins from the Ras signaling pathway (http://www.genome.jp/dbget-bin/www_bget?pathway:bta04014), and Wnt proteins from the Wnt signaling pathway (http://www.genome.jp/dbget-bin/www_bget?pathway:bta04310).

Phospholipase D from the phospholipase D signaling path way is an essential enzyme for the production of phosphatidic acid (http://www.genome.jp/dbget-bin/www_bget?pathway:bta04310), a key intermediate in milk fat synthesis during lactation [25]. The Focal adhesion and Gap junction pathways receive and send signals that affect the motility, proliferation, differentiation, metabolic transport, apoptosis, and tissue homeostasis of ovarian follicle and mammary gland cells [21]. The cyclic guanosine monophosphate from the cGMP-PKG signaling pathway involved in the activation and regulation of protein kinase G in smooth muscle cells to promote their relaxation (http://www.genome.jp/dbget-bin/www_bget?pathway:bta04022). The ceramide and sphingosine-1-phosphate from the sphingolipid signaling pathway acts an as a regulator of cell responses to stress (http://www.genome.jp/dbget-bin/www_bget?pathway:bta04071). The oxytocin hormone (oxytocin signaling pathway), produced by the hypothalamus, stimulates the contraction of mammary gland myoepithelial cells, causing milk to be ejected into the ducts, and cisterns during milking (http://www.genome.jp/dbget-bin/www_bget?pathway:bta04921).

Cellular processes influenced MY, FY, and AFC through a large number of genes located in multiple pathways, each having a small effect and explaining a small percentage of genetic variation for these traits. However, the combined effect of all genes in all enriched cellular pathways explained a noticeable amount of genetic variation. Therefore, the combined effect of all cellular processes for MY, FY, and AFC could potentially be considered as a functional genomic selection target within each trait in this population.

#### Nervous system

The nervous system pathways include glutamatergic synapse, GABAergic synapse, and dopaminergic synapse pathways involved in brain remodeling. There were 70 genes in these three pathways, and they together explained 0.78% of the genetic variance for MY, 0.66% for FY, and 0.78% for AFC (Table 1), and their associated SNP had a total predicted value of −4.1918 for MY, −0.0938 for FY, and 0.0039 for AFC (Table 2). These three pathways are involved in the onset of puberty, which in turn determines AFC. Changes in the concentration of gonadotropin-releasing hormone (GnRH) trigger of the onset of puberty. Glutamate from the glutamatergic synapse pathway and gamma-aminobutiyric acid from the GABAergic synapse pathway stimulate the production of GnRH whereas dopamine from the dopaminergic synapse inhibits it [26,27]. The GnRH stimulates the secretion of gonadotropins from the pituitary gland (luteinizing and follicle-stimulating hormones) involved in the development of follicles, ovulation and the production of estrogen and progesterone. Fortes et al [28] provided evidence for the involvement of genes from the glutamatergic synapse and GABAergic synapse pathways in the attainment of puberty in beef cattle.

#### Digestive system

The only significant pathway in the digestive system category was the pancreatic secretion pathway. This pathway was 27 genes and they together explained 0.27% of the genetic variance for MY, 0.29% for FY, and 0.32% for AFC (Table 1), and the total predicted value of the associated SNP were −0.9985 for MY, −0.0260 for FY, and −0.0006 for AFC (Table 2). The digestive system pathway was also found to be associated with milk production traits in Holstein [7]. Pancreatic enzymes (lipases, amylases, proteases) from the pancreatic secretion pathway are important for the digestion and absorption of nutrients (carbohydrates, proteins, fats, vitamins) in the small intestine. Thus, genes involved in the pancreatic pathway likely influenced differences in MY, FY, and AFC among animals in the Thai multibreed dairy population. Heifers that digested and absorbed nutrients more efficiently would be expected to have faster growth rates, achieve puberty earlier, have higher conception rates and produce more milk than less efficient heifers. Thus, heifers in the Thai multibreed dairy population that digested and absorbed nutrients from local roughages, concentrate and byproducts of agricultural efficiently likely had lower AFC than less efficient heifers.

#### Environmental adaptation

The circadian entrainment pathway was the only significant pathway in the environmental adaptation category. This pathway contained 30 genes that explained 0.38% of the genetic variance for MY, 0.36% for FY, and 0.37% for AFC (Table 1), and the sum of the predicted values of the SNP associated with these genes was −1.0546 for MY, −0.0425 for FY, and −0.0020 for AFC (Table 2). The circadian entrainment pathway is important for animal adaptation to number of daylight hours, temperature and humidity [29]. The cyclic adenosine monophosphate (cAMP) response element-binding protein from the circadian entrainment pathway regulates the circadian clock (http://www.genome.jp/dbget-bin/www_bget?pathway:bta04713). The circadian clock is influenced by the length of photoperiod [30], which in turn influences the activity of multiple hormones (estrogen, progesterone, placental lactogen, prolactin, leptin, cortisol) that affect metabolites (glucose, amino acids, free fatty acids, triglycerides) received by cells of the mammary gland [31]. Dairy cows in Thailand are exposed directly to day-length changes because farmers house cows in open barns. Thus, it is not surprising that genes involved in the circadian entrainment pathway explained a significant portion of the genetic variation for MY and FY in the Thai multibreed dairy population.

### Protein-protein interaction network analysis

**Figure 4:**
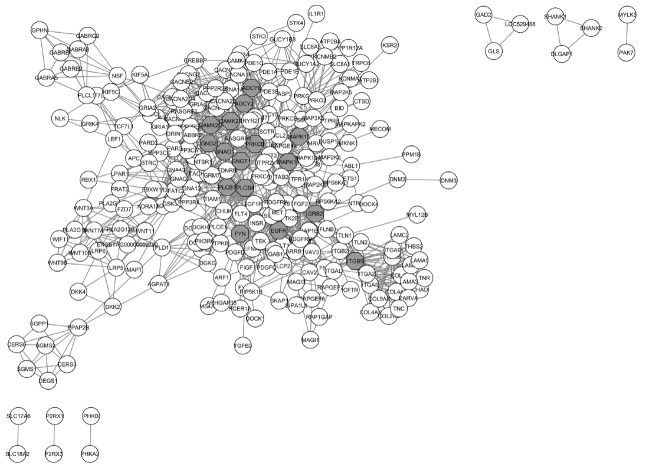
Protein-protein interaction (PPI) network of genes involved in significant pathways. Gray nodes represent genes with large numbers of connections with other genes in the PPI network.

**Figure 5:**
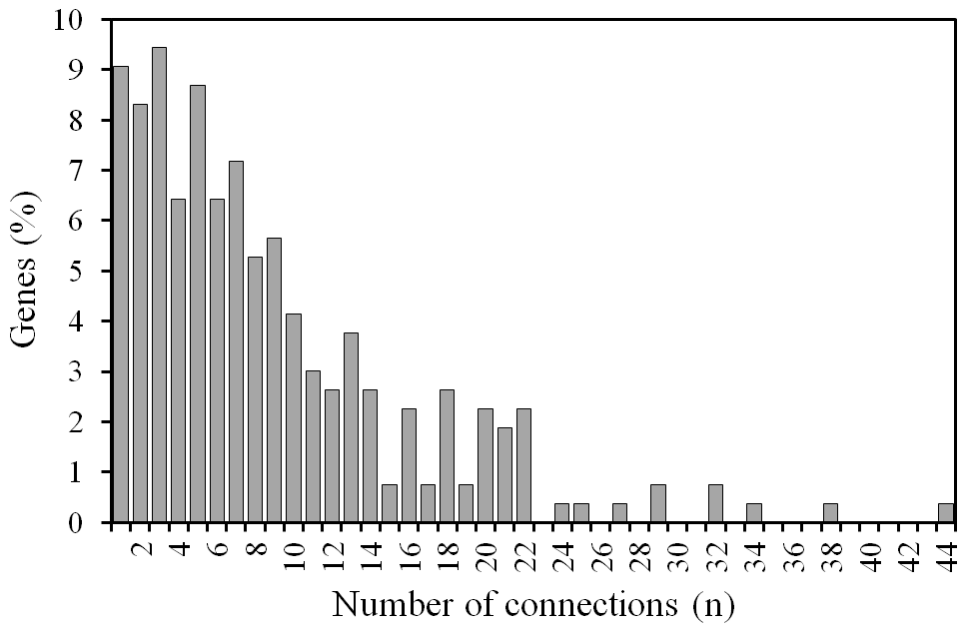
Distribution of genes associated with milk yield, fat yield, and age at first calving by number of connections in the protein-protein interaction network.

The PPI network for MY, FY, and AFC contained 265 nodes (i.e., genes) connected via 1,158 edges (Figure 4). Approximately 90% of the genes had two or more connections (Figure 5). The preponderance of multiple interactions among genes in the PPI network indicated that this was a highly interconnected network where most genes affected the expression of other genes relevant to MY, FY, and AFC. The number of connections per node ranged from 1 to 44 and the number of pathways fluctuated between 1 and 15 (Supplementary File S1). The PPI network for MY, FY, and AFC showed a dense center with highly interconnected genes (Figure 4). Genes in the PPI network explained 2.28% of the genetic variance for MY, 2.26% for FY, and 2.12% for AFC (Supplementary File S1). Thus, genes in the PPI network explained an average of 86.3% of the genetic variation as genes present in significantly enriched pathways (86.7% for MY, 87.2% for FY, and 85.2% for AFC). The sum of the predicted SNP values of the 265 genes in the PPI network was −5.6150 for MY, −0.1404 for FY, and 0.0067 for AFC (Supplementary File S2). As with explained genetic variances, these sums of predicted values for PPI genes were similar to those obtained for the 303 genes in the significantly enriched pathways (Table 2). All genes in the PPI network were also involved in one or more enriched pathways in Table 1. Thus, the 265 genes in the PPI network were a subset of the 303 genes in the set of enriched pathways, meaning that 87.5 of enriched pathway genes were represented in the PPI network. Thus, the 14% lower amount of genetic variation explained by PPI genes was due to a 12.5% lower number of genes than those present in the enriched pathways in Table 1.

**Figure 6:**
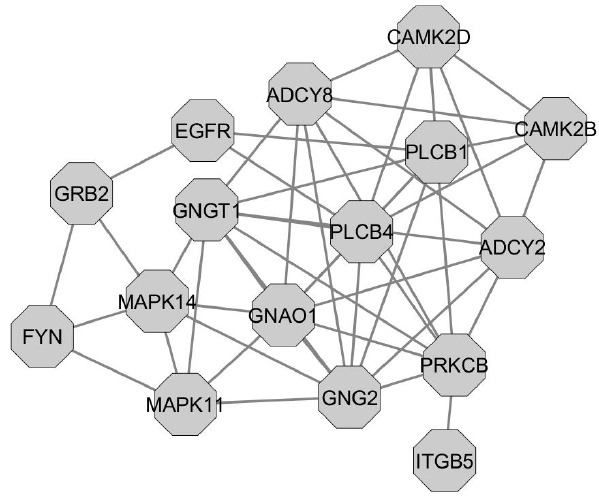
Genes with large numbers of connections in the protein-protein interaction network of the Thai multibreed population.

Figure 6 show a subset of the most represented genes in the PPI network for MY, FY, and AFC (16 genes with a minimum of 22 connections and 1 pathway). The protein kinase C beta (*PRKCB*) gene had the largest number of significantly enriched pathways for MY, FY, and AFC (14) and accounted for 0.012% of the genetic variance for MY, 0.009% for FY and 0.016% for AFC (Supplementary File S1). This gene participated in 9 biological process pathways, 3 nervous system pathways, digestive system pathway and environmental adaptation pathway. *PRKCB* codes for protein kinase C beta that is involved in diverse cellular signaling pathways (http://www.genecards.org/cgi-bin/carddisp.pl?gene=PRKCB&keywords=PRKCB). Further, *PRKCB* is also involved in the circadian entrainment pathway. This pathway contributes to the adaptation of organisms to their environment [29]. A positive influence of *PRKCB* on body temperature regulation during climate stress was reported in Angus and Simmental cattle [32]. Higher MY and fat percentages were observed in Holstein that were better adapted to climatic heat stress [33]. The predicted value of the set of SNP associated with *PRKCB* was 0.226 for MY, 0.005 for FY, and −0.002 for AFC (Supplementary File S2). These predicted SNP values indicate that the second *PRKCB* allele would result in higher MY and FY as well as shorter AFC in cows from the Thai multibreed dairy population, whereas the first *PRKCB* allele would have the opposite effect.

The phospholipase C beta 1 ( *PLCB1*), *PLCB4*, adenylate cyclase 2 (*ADCY2*), *ADCY8*, calcium/calmodulin dependent protein kinase II beta (*CAMK2B*), *CAMK2D*, mitogen-activated protein kinase 11 (*MAPK11*), *MAPK14*, epidermal growth factor receptor (*EGFR*), growth factor receptor bound protein 2 (*GRB2*), Fyn proto-oncogene, Src family tyrosine kinase (*FYN*), and integrin subunit beta 5 (*ITGB5*) genes were involved in 12 significantly enriched pathways and accounted for 0.126% of the genetic variance for MY, 0.109% for FY, and 0.197% for AFC (Supplementary File S1). The predicted values of the set of SNP associated with these genes was −0.739 for MY, −0.026 for FY, and 0.001 for AFC. These predicted SNP values indicated that the subset of 16 PPI second alleles would decrease MY, FY, and increase AFC, whereas the subset of 16 PPI first alleles would increase MY and FY, but decrease AFC. These genes participated in 8 cellular process pathways (such as MAPK signaling, Ras signaling, Wnt signaling) related to the development of ovarian follicles and cells from the mammary gland [6,21]. *PLCB1* and *PLCB4* code for phospholipase C beta 1 to 4 that function as signal transducers for the transmission of extracellular signals to multiple intracellular targets (http://www.genecards.org/cgi-bin/carddisp.pl?gene=PLCB1&keywords=PLCB1; http://www.genecards.org/cgi-bin/carddisp.pl?gene=PLCB4&keywords=PLCB4). *ADCY2* and *ADCY8* code for adenylyl cyclase type 2 and 8 that act as catalysts for the formation of cAMP which is involved in many cellular processes (http://www.genecards.org/cgi-bin/carddisp.pl?gene=ADCY2&keywords=ADCY2; http://www.genecards.org/cgi-bin/carddisp.pl?gene=ADCY8&keywords=ADCY8). *CAMK2B* and *CAMK2D* code for calcium/calmodulin-dependent protein kinases that function as mediators of calcium signaling in cells (http://www.genecards.org/cgi-bin/carddisp.pl?gene=CAMK2B&keywords=CAMK2B; http://www.genecards.org/cgi-bin/carddisp.pl?gene=CAMK2D&keywords=CAMK2D). *MAPK11* and *MAPK14* code for p38 mitogen-activated protein kinases 11 to 14 that function as mediators of the cellular response to external signals [34]. *EGFR* codes for epidermal growth factor receptor that act as a receptor for the growth factor (http://www.genecards.org/cgi-bin/carddisp.pl?gene=EGFR&keywords=EGFR). *GRB2* codes for a growth factor receptor-bound protein that functions as a signal transducer (http://www.genecards.org/cgi-bin/carddisp.pl?gene=GRB2&keywords=GRB2). *FYN* codes for protein-tyrosine kinase that acts as an activator of molecular signals (http://www.genecards.org/cgi-bin/carddisp.pl?gene=FYN&keywords=FYN). *ITGB5* codes for integrin beta type 5 that functions as a receptor for fibronectin (http://www.genecards.org/cgi-bin/carddisp.pl?gene=ITGB5&keywords=ITGB5), which regulates cell proliferation and differentiation during the development of ovarian follicles and mammary gland cells.

The G protein subunit gamma 2 ( *GNG2*), G protein subunit gamma transducin 1 (*GNGT1*), and G protein subunit alpha O1 (*GNAO1*) genes were involved in 6 significantly enriched pathways and accounted for 0.040% of the genetic variance for MY, 0.027% for FY, and 0.020% for AFC (Supplementary File S1). The predicted values of the set of SNP associated with these genes were −0.177 for MY, 0.001 for FY, and −0.002 for AFC (Supplementary File S2). Thus, the combined effect of the three second alleles from these genes would decrease MY, increase FY, and decrease AFC, and the set of first alleles of these genes would have the opposite effect. These three genes involved in the glutamatergic, GABAergic and dopaminergic synapse pathways. These three pathways are involved in the onset of puberty [28]. Lastly, *GNAO1* participates in the development of ovarian follicles [21].

Pathway enrichment and PPI network analyses indicated that MY, FY, and AFC of animals in the Thai multibreed dairy population were influenced by sets of genes that were important for cellular processes, nervous and digestive systems and environmental adaptation. Cellular processes were involved with the largest number of biological pathways and PPI among genes associated with MY, FY, and AFC. This likely occurred because cellular processes are important for fundamental cell activities related to the development of cells from the mammary gland and the development of ovarian follicles. Although individual genes or biological pathways explained a small fraction of the genetic variance for MY, FY, and AFC, the combined effect of all genes in all enriched biological pathways and the PPI network explained a substantially larger amount of the genetic variance for these traits. Thus, the set of SNP associated with the enriched pathways and the PPI network in this study could be considered as specific genomic selection targets to help increase MY, FY, and decrease AFC in the Thai multibreed dairy population. However, because the amount of explained genetic variation for each trait was a minor fraction of their total, these studies need to continue with the ultimate goal of accounting for most of the genetic variation due to biological processes in the Thai multibreed dairy population. It should be kept in mind that size and direction of the predicted SNP values here will likely differ in other dairy populations due to breed composition and environmental conditions (climate, management, nutrition, and health care) and will also likely differ over time as population characteristics and environmental conditions change.

## Figures and Tables

**Table 1:** Percent of genetic variance for milk yield (MY), fat yield (FY), and age at first calving (AFC) explained by single nucleotide polymorphism located inside or within 2,500 bp of genes present in significantly enriched pathways

Category	Pathway	p-value	Number of genes (n)	Genetic variance (%)		

				MY	FY	AFC
Cellular processes			266	2.2408	2.2867	2.1220
	Rap1 signaling	8.9×10^−8^	63	0.5113	0.6292	0.6437
	Calcium signaling	1.3×10^−7^	58	0.5018	0.5157	0.5381
	Phospholipase D signaling	2.8×10^−6^	47	0.4691	0.4361	0.4657
	Focal adhesion	1.6×10^−5^	55	0.4087	0.3868	0.4621
	MAPK signaling	0.0002	62	0.4847	0.523	0.4807
	Ras signaling	0.0075	55	0.3982	0.4333	0.4639
	Wnt signaling	0.0077	38	0.3062	0.3016	0.2848
	cGMP-PKG signaling	0.0085	41	0.4702	0.483	0.4668
	Sphingolipid signaling	0.014	32	0.2295	0.2561	0.3181
	Oxytocin signaling	0.016	38	0.3528	0.3538	0.376
	Gap junction	0.021	26	0.3223	0.2973	0.3667
Nervous system			70	0.7829	0.6567	0.7825
	Glutamatergic synapse	1.9×10^−8^	42	0.4857	0.4409	0.4886
	Dopaminergic synapse	0.0036	36	0.4004	0.3415	0.4037
	GABAergic synapse	0.011	26	0.3182	0.2633	0.2692
Digestive system			27	0.2748	0.2902	0.3216
	Pancreatic secretion	0.03	27	0.2748	0.2902	0.3216
Environmental adaptation			30	0.3818	0.3593	0.3672
	Circadian entrainment	0.0018	30	0.3818	0.3593	0.3672
Total			303	2.6282	2.5916	2.4893

**Table 2:** Predicted value of SNP located inside or within 2,500 bp of genes associated with milk yield (MY), fat yield (FY), and age at first calving (AFC) present in significantly enriched pathways

Category	Pathway	p-value	Number of genes (n)	Predicted SNP value		

				MY	FY	AFC
Cellular processes			266	−3.7513	−0.1524	0.0128
	Rap1 signaling	8.9×10−8	63	−0.9683	−0.0715	0.0036
	Calcium signaling	1.3×10−7	58	−2.5956	−0.0520	0.0005
	Phospholipase D signaling	2.8×10−6	47	−2.7299	−0.0919	0.0072
	Focal adhesion	1.6×10−5	55	−3.5352	−0.0795	−0.0027
	MAPK signaling	0.0002	62	−0.0853	−0.0382	0.0033
	Ras signaling	0.0075	55	−1.1262	−0.0448	0.0025
	Wnt signaling	0.0077	38	−0.2874	−0.0133	0.0017
	cGMP-PKG signaling	0.0085	41	−0.5336	−0.0534	0.0037
	Sphingolipid signaling	0.014	32	0.5767	0.0287	0.0056
	Oxytocin signaling	0.016	38	−0.9332	−0.0438	−0.0022
	Gap junction	0.021	26	−1.0156	−0.0477	0.0017
Nervous system			70	−4.1918	−0.0938	0.0039
	Glutamatergic synapse	1.9×10−8	42	−2.0444	−0.0804	−0.0032
	Dopaminergic synapse	0.0036	36	−1.2675	−0.0328	0.0002
	GABAergic synapse	0.011	26	−2.5585	−0.0434	0.0041
Digestive system			27	−0.9985	−0.0260	−0.0006
	Pancreatic secretion	0.03	27	−0.9985	−0.0260	−0.0006
Environmental adaptation			30	−1.0546	−0.0425	−0.0020
	Circadian entrainment	0.0018	30	−1.0546	−0.0425	−0.0020
Total			303	−5.0430	−0.1573	0.0077

SNP, single nucleotide polymorphism.
