# Supplementary material for: Pathway enrichment and protein interaction network analysis for milk yield, fat yield and age at first calving in a Thai multibreed dairy population
Source: Asian-Australas J Anim Sci. 2018 Jul 26;32(4):508–18. doi: 10.5713/ajas.18.0382 (PMC6409460; doi:10.5713/ajas.18.0382)
Supplement: Supplementary file 1 [file ajas-18-0382-supplementary.pdf]

**Supplementary Table S1** *Number of the SNP markers with genetic variance larger than or equal to 0.001% for milk yield, fat yield, and age at first calving by distance between SNP genotypes and genes in the NCBI database.*

| Distance between SNP and gene | Milk yield | Fat yield | Age at first calving |
|---|---|---|---|
| Inside gene | 10,263 | 10,119 | 10,149 |
| ≤ 2500 bp | 1,390 | 1,406 | 1,360 |
| 2500 bp < distance ≤ 5000 bp | 855 | 848 | 878 |
| 5000 bp < distance ≤ 25 000 bp | 3,726 | 3,739 | 3,713 |
| distance > 25 000 bp | 10,342 | 10,287 | 10,555 |
| **Total** | **26,576** | **26,399** | **26,655** |

**Supplementary Table S2** *Number of genes and total genetic variance for milk yield (MY), fat yield (FY), and age at first calving (AFC) per chromosome identified by SNP markers inside genes or within 2500 bp of genes in the Thai multibreed population.*

| Chromosome | Number of genes (n) | | | Genetic variance (%) | | |
|---|---|---|---|---|---|---|
| | MY | FY | AFC | MY | FY | AFC |
| 1 | 299 | 296 | 323 | 1.46 | 1.37 | 1.69 |
| 2 | 322 | 307 | 292 | 1.58 | 1.49 | 1.53 |
| 3 | 342 | 365 | 347 | 1.60 | 1.70 | 1.66 |
| 4 | 286 | 282 | 282 | 1.91 | 1.73 | 2.01 |
| 5 | 393 | 361 | 403 | 2.02 | 2.07 | 1.96 |
| 6 | 256 | 236 | 241 | 1.40 | 1.27 | 1.39 |
| 7 | 326 | 342 | 341 | 1.57 | 1.53 | 1.50 |
| 8 | 271 | 272 | 264 | 1.58 | 1.49 | 1.48 |
| 9 | 243 | 230 | 230 | 1.40 | 1.45 | 1.29 |
| 10 | 280 | 304 | 309 | 1.75 | 1.74 | 1.81 |
| 11 | 327 | 349 | 339 | 1.77 | 2.09 | 1.70 |
| 12 | 162 | 168 | 157 | 1.20 | 1.45 | 1.39 |
| 13 | 238 | 263 | 248 | 1.33 | 1.29 | 1.40 |
| 14 | 195 | 196 | 185 | 1.36 | 1.40 | 1.24 |
| 15 | 299 | 295 | 299 | 1.70 | 1.54 | 1.66 |
| 16 | 223 | 237 | 244 | 1.38 | 1.39 | 1.28 |
| 17 | 224 | 224 | 214 | 1.39 | 1.29 | 0.96 |
| 18 | 300 | 299 | 286 | 1.22 | 1.35 | 1.25 |
| 19 | 325 | 317 | 291 | 1.67 | 1.64 | 1.46 |
| 20 | 141 | 137 | 131 | 0.80 | 0.64 | 0.72 |
| 21 | 156 | 138 | 151 | 0.86 | 0.75 | 0.79 |
| 22 | 138 | 134 | 138 | 0.73 | 0.73 | 0.83 |
| 23 | 140 | 157 | 154 | 0.82 | 0.83 | 0.70 |
| 24 | 118 | 118 | 125 | 0.65 | 0.62 | 0.72 |
| 25 | 169 | 177 | 162 | 0.84 | 0.84 | 0.83 |

| Chromosome | Number of genes (n) | | | Genetic variance (%) | | |
|---|---|---|---|---|---|---|
| | **MY** | **FY** | **AFC** | **MY** | **FY** | **AFC** |
| 26 | 153 | 156 | 159 | 0.75 | 0.81 | 0.87 |
| 27 | 105 | 109 | 106 | 0.66 | 0.58 | 0.58 |
| 28 | 146 | 131 | 141 | 0.88 | 0.85 | 0.95 |
| 29 | 176 | 173 | 171 | 0.91 | 1.01 | 0.93 |
| X | 177 | 186 | 189 | 0.87 | 0.87 | 0.91 |
| **Total** | **6,930** | **6,959** | **6,922** | **38.04** | **37.81** | **37.50** |

**Supplementary Table S3** *Number of genes and total genetic variance for milk yield (MY), fat yield (FY) and age at first calving (AFC) per chromosome in the Thai multibreed population.*

| Chromosome | Number of genes (n) | Genetic variance (%) | | |
|---|---|---|---|---|
| | | MY | FY | AFC |
| 1 | 149 | 1.026 | 0.917 | 1.075 |
| 2 | 176 | 1.103 | 1.113 | 1.193 |
| 3 | 159 | 1.073 | 1.094 | 0.964 |
| 4 | 152 | 1.402 | 1.356 | 1.490 |
| 5 | 192 | 1.406 | 1.508 | 1.239 |
| 6 | 135 | 0.987 | 0.925 | 0.957 |
| 7 | 147 | 0.883 | 0.928 | 0.826 |
| 8 | 142 | 1.165 | 1.096 | 0.971 |
| 9 | 118 | 0.951 | 1.001 | 0.873 |
| 10 | 158 | 1.274 | 1.243 | 1.129 |
| 11 | 174 | 1.191 | 1.436 | 1.110 |
| 12 | 94 | 1.014 | 1.211 | 1.109 |
| 13 | 118 | 0.919 | 0.862 | 0.959 |
| 14 | 107 | 1.015 | 1.056 | 0.924 |
| 15 | 137 | 1.088 | 0.971 | 1.060 |
| 16 | 117 | 0.976 | 0.964 | 0.773 |
| 17 | 111 | 0.990 | 0.881 | 0.627 |
| 18 | 108 | 0.630 | 0.754 | 0.712 |
| 19 | 152 | 1.040 | 1.043 | 0.942 |
| 20 | 81 | 0.605 | 0.503 | 0.495 |
| 21 | 65 | 0.567 | 0.515 | 0.488 |
| 22 | 67 | 0.533 | 0.565 | 0.522 |
| 23 | 69 | 0.563 | 0.537 | 0.422 |
| 24 | 62 | 0.446 | 0.454 | 0.436 |
| 25 | 74 | 0.581 | 0.529 | 0.566 |
| 26 | 77 | 0.520 | 0.583 | 0.592 |
| 27 | 55 | 0.439 | 0.363 | 0.419 |
| 28 | 74 | 0.683 | 0.672 | 0.626 |
| 29 | 83 | 0.599 | 0.696 | 0.670 |
| X | 83 | 0.556 | 0.543 | 0.526 |
| **Total** | **3436** | **26.223** | **26.317** | **24.693** |

**Supplementary File S1** *Number of connections, number of pathways and percent of genetic variance for milk yield (MY), fat yield (FY), and age at first calving (AFC) explained by single nucleotide polymorphism located inside or within 2,500 bp of genes in the Thai multibreed population.*

| Gene name | Gene description | Number of Connections (n) | Number of pathways (n) | Genetic variance (%) | | |
|---|---|---|---|---|---|---|
| | | | | MY | FY | AFC |
| PLCB1 | phospholipase C beta 1 | 44 | 12 | 0.0475 | 0.0423 | 0.0674 |
| GNG2 | G protein subunit gamma 2 | 38 | 5 | 0.0296 | 0.0158 | 0.0067 |
| PLCB4 | phosphoinositide phospholipase C | 34 | 12 | 0.0066 | 0.0077 | 0.0227 |
| CAMK2B | calcium/calmodulin dependent protein kinase II beta | 32 | 5 | 0.0012 | 0.0014 | 0.0062 |
| CAMK2D | calcium/calmodulin dependent protein kinase II delta | 32 | 5 | 0.0199 | 0.0067 | 0.0073 |
| MAPK14 | mitogen-activated protein kinase 14 | 29 | 4 | 0.0018 | 0.0026 | 0.0087 |
| GRB2 | growth factor receptor bound protein 2 | 29 | 5 | 0.0107 | 0.0032 | 0.0104 |
| ITGB5 | integrin subunit beta 5 | 27 | 1 | 0.0012 | 0.0025 | 0.0018 |
| PRKCB | protein kinase C beta | 25 | 14 | 0.0120 | 0.0091 | 0.016 |
| FYN | FYN proto-oncogene, Src family tyrosine kinase | 24 | 3 | 0.0011 | 0.0033 | 0.0129 |
| GNGT1 | G protein subunit gamma transducin 1 | 22 | 5 | 0.0061 | 0.0057 | 0.0026 |
| ADCY8 | adenylate cyclase 8 | 22 | 10 | 0.0201 | 0.0127 | 0.0167 |
| ADCY2 | adenylate cyclase 2 | 22 | 10 | 0.0097 | 0.0137 | 0.0215 |
| GNAO1 | G protein subunit alpha o1 | 22 | 6 | 0.0047 | 0.0057 | 0.0102 |
| MAPK11 | mitogen-activated protein kinase 11 | 22 | 4 | 0.0019 | 0.0027 | 0.0019 |
| EGFR | epidermal growth factor receptor | 22 | 8 | 0.0041 | 0.0098 | 0.0194 |
| ADCY5 | adenylate cyclase 5 | 21 | 10 | 0.0044 | 0.0011 | 0.0015 |
| ADCY7 | adenylate cyclase 7 | 21 | 10 | 0.0050 | 0.0149 | 0.0031 |
| GNAQ | G protein subunit alpha q | 21 | 10 | 0.0052 | 0.0063 | 0.0066 |

| Gene name | Gene description | Number of Connections (n) | Number of pathways (n) | Genetic variance (%) | | |
|---|---|---|---|---|---|---|
| | | | | MY | FY | AFC |
| RAP1B | RAP1B, member of RAS oncogene family | 21 | 5 | 0.0040 | 0.012 | 0.0049 |
| IRS1 | insulin receptor substrate 1 | 21 | 1 | 0.0123 | 0.0076 | 0.0043 |
| PRKCA | protein kinase C alpha | 20 | 15 | 0.0096 | 0.0216 | 0.0064 |
| ITGA6 | integrin subunit alpha 6 | 20 | 1 | 0.0076 | 0.0043 | 0.003 |
| ITGA9 | integrin subunit alpha 9 | 20 | 1 | 0.0072 | 0.0162 | 0.0167 |
| ITGA8 | integrin subunit alpha 8 | 20 | 1 | 0.0312 | 0.017 | 0.0187 |
| PTEN | phosphatase and tensin homolog | 20 | 2 | 0.0023 | 0.0043 | 0.0031 |
| AKT3 | AKT serine/threonine kinase 3 | 20 | 8 | 0.0038 | 0.0042 | 0.01 |
| ITGA2 | integrin subunit alpha 2 | 19 | 1 | 0.0014 | 0.0019 | 0.0101 |
| IGF1R | insulin like growth factor 1 receptor | 19 | 3 | 0.0069 | 0.0027 | 0.0048 |
| RASGRF2 | RAS protein-specific guanine nucleotide-releasing factor 2 | 18 | 2 | 0.0059 | 0.0022 | 0.003 |
| PPP2R5E | protein phosphatase 2 regulatory subunit B'epsilon | 18 | 2 | 0.007 | 0.0054 | 0.0034 |
| PDGFRA | platelet derived growth factor receptor alpha | 18 | 7 | 0.001 | 0.0023 | 0.0032 |
| PDGFRB | platelet derived growth factor receptor beta | 18 | 7 | 0.0039 | 0.0078 | 0.0103 |
| MAPK10 | mitogen-activated protein kinase 10 | 18 | 6 | 0.0058 | 0.0041 | 0.0168 |
| CACNG2 | calcium voltage-gated channel auxiliary subunit gamma 2 | 18 | 2 | 0.0043 | 0.0039 | 0.01 |
| SOS1 | SOS Ras/Rac guanine nucleotide exchange factor 1 | 18 | 5 | 0.0046 | 0.005 | 0.0042 |
| PRKG2 | protein kinase, cGMP-dependent, type II | 17 | 3 | 0.0116 | 0.0106 | 0.0022 |
| PRKG1 | protein kinase, cGMP-dependent, type I | 17 | 3 | 0.0366 | 0.0371 | 0.0341 |
| ADCY1 | adenylate cyclase 1 | 16 | 10 | 0.0021 | 0.0025 | 0.0035 |
| LPAR3 | lysophosphatidic acid receptor 3 | 16 | 2 | 0.0014 | 0.004 | 0.0027 |
| GRM1 | glutamate receptor, metabotropic 1 | 16 | 4 | 0.0152 | 0.0189 | 0.0128 |
| PLCE1 | phospholipase C epsilon 1 | 16 | 3 | 0.0068 | 0.0094 | 0.0052 |
| INSR | insulin receptor | 16 | 4 | 0.0132 | 0.0303 | 0.0111 |

| Gene name | Gene description | Number of Connections (n) | Number of pathways (n) | Genetic variance (%) | | |
|---|---|---|---|---|---|---|
| | | | | MY | FY | AFC |
| SHC3 | SHC adaptor protein 3 | 16 | 3 | 0.0029 | 0.0034 | 0.0065 |
| CACNG4 | calcium voltage-gated channel auxiliary subunit gamma 4 | 15 | 2 | 0.008 | 0.0014 | 0.0064 |
| GSK3B | glycogen synthase kinase 3 beta | 15 | 3 | 0.0119 | 0.0058 | 0.0014 |
| PPP2R2B | protein phosphatase 2 regulatory subunit Bbeta | 14 | 2 | 0.0075 | 0.0015 | 0.002 |
| CACNB2 | calcium voltage-gated channel auxiliary subunit beta 2 | 14 | 2 | 0.0096 | 0.0124 | 0.0079 |
| FLT1 | fms related tyrosine kinase 1 | 14 | 3 | 0.0092 | 0.0065 | 0.0016 |
| GRIA3 | glutamate ionotropic receptor AMPA type subunit 3 | 14 | 3 | 0.0118 | 0.0164 | 0.0085 |
| MET | MET proto-oncogene, receptor tyrosine kinase | 14 | 3 | 0.0015 | 0.0026 | 0.0041 |
| GRIA1 | glutamate receptor, ionotropic, AMPA1 | 14 | 3 | 0.0235 | 0.0096 | 0.0123 |
| TEK | TEK receptor tyrosine kinase | 14 | 2 | 0.007 | 0.0067 | 0.0017 |
| RASGRP3 | RAS guanyl releasing protein 3 | 13 | 3 | 0.0055 | 0.0028 | 0.0024 |
| PLD1 | phospholipase D1, phosphatidylcholine-specific | 13 | 4 | 0.0042 | 0.0015 | 0.0339 |
| FGF19 | fibroblast growth factor 19 | 13 | 3 | 0.009 | 0.0025 | 0.0032 |
| EFNA5 | ephrin A5 | 13 | 2 | 0.0085 | 0.0062 | 0.0063 |
| CACNB4 | calcium voltage-gated channel auxiliary subunit beta 4 | 13 | 2 | 0.0099 | 0.0038 | 0.0034 |
| GNA12 | G protein subunit alpha 12 | 13 | 4 | 0.0055 | 0.0023 | 0.0042 |
| FGF2 | BFGF | 13 | 3 | 0.0081 | 0.0063 | 0.0014 |
| TLN1 | talin 1 | 13 | 2 | 0.0017 | 0.0029 | 0.0032 |
| GRIA2 | glutamate ionotropic receptor AMPA type subunit 2 | 13 | 3 | 0.0013 | 0.0014 | 0.0105 |
| FLT4 | fms related tyrosine kinase 4 | 13 | 3 | 0.0023 | 0.0076 | 0.0164 |
| PDE3B | phosphodiesterase 3B | 12 | 1 | 0.0144 | 0.0074 | 0.0013 |
| EDNRB | endothelin receptor type B | 12 | 2 | 0.0012 | 0.0015 | 0.0045 |
| CACNA1A | calcium voltage-gated channel subunit alpha1 A | 12 | 5 | 0.0243 | 0.0255 | 0.0143 |

| Gene name | Gene description | Number of Connections (n) | Number of pathways (n) | Genetic variance (%) | | |
|---|---|---|---|---|---|---|
| | | | | MY | FY | AFC |
| GNA13 | G protein subunit alpha 13 | 12 | 3 | 0.0037 | 0.0049 | 0.0029 |
| GRIA4 | glutamate ionotropic receptor AMPA type subunit 4 | 12 | 3 | 0.0342 | 0.0257 | 0.029 |
| ITGB2 | integrin subunit beta 2 | 12 | 1 | 0.0036 | 0.004 | 0.0053 |
| ANGPT1 | angiopoietin 1 | 12 | 2 | 0.0248 | 0.0249 | 0.0025 |
| PDE3A | phosphodiesterase 3A | 11 | 1 | 0.012 | 0.0143 | 0.0078 |
| CACNA2D4 | calcium voltage-gated channel auxiliary subunit alpha2delta 4 | 11 | 2 | 0.0063 | 0.0095 | 0.0013 |
| CACNA2D3 | calcium voltage-gated channel auxiliary subunit alpha2delta 3 | 11 | 2 | 0.0302 | 0.0238 | 0.0295 |
| FN1 | fibronectin 1 | 11 | 1 | 0.0022 | 0.0039 | 0.001 |
| BCL2 | BCL2, apoptosis regulator | 11 | 2 | 0.0099 | 0.0132 | 0.0015 |
| GRIN2B | glutamate ionotropic receptor NMDA type subunit 2B | 11 | 5 | 0.0137 | 0.0243 | 0.0048 |
| GRIN1 | glutamate ionotropic receptor NMDA type subunit 1 | 11 | 5 | 0.002 | 0.004 | 0.0069 |
| LRP6 | LDL receptor related protein 6 | 11 | 1 | 0.0026 | 0.0082 | 0.0066 |
| CACNG5 | calcium voltage-gated channel auxiliary subunit gamma 5 | 10 | 2 | 0.0016 | 0.008 | 0.0013 |
| FGF12 | fibroblast growth factor 12 | 10 | 3 | 0.015 | 0.0202 | 0.0198 |
| PDGFD | platelet derived growth factor D | 10 | 5 | 0.0282 | 0.0151 | 0.0331 |
| PDGFC | platelet derived growth factor C | 10 | 5 | 0.0078 | 0.003 | 0.0156 |
| CASP3 | caspase 3, apoptosis-related cysteine peptidase | 10 | 1 | 0.0017 | 0.0025 | 0.0011 |
| RAPGEF4 | Rap guanine nucleotide exchange factor 4 | 10 | 2 | 0.0036 | 0.0027 | 0.0089 |
| FLNB | filamin B | 10 | 2 | 0.0087 | 0.0081 | 0.0044 |
| LRP5 | LDL receptor related protein 5 | 10 | 1 | 0.0075 | 0.0178 | 0.0046 |
| FIGF | c-fos induced growth factor | 10 | 3 | 0.0102 | 0.0121 | 0.0035 |
| WNT3A | Wnt family member 3A | 10 | 1 | 0.004 | 0.0021 | 0.0022 |

| Gene name | Gene description | Number of Connections (n) | Number of pathways (n) | Genetic variance (%) | | |
|---|---|---|---|---|---|---|
| | | | | MY | FY | AFC |
| GAB1 | GRB2 associated binding protein 1 | 10 | 2 | 0.0057 | 0.0092 | 0.002 |
| TACR1 | tachykinin receptor 1 | 9 | 1 | 0.0177 | 0.0214 | 0.0078 |
| TACR3 | tachykinin receptor 3 | 9 | 1 | 0.0049 | 0.0049 | 0.004 |
| NTSR1 | neurotensin receptor 1 | 9 | 1 | 0.0015 | 0.0023 | 0.0022 |
| NSF | N-ethylmaleimide sensitive factor, vesicle fusing ATPase | 9 | 1 | 0.0055 | 0.0075 | 0.0044 |
| PDE1A | phosphodiesterase 1A, calmodulin-dependent | 9 | 1 | 0.0055 | 0.0014 | 0.0017 |
| PDE1C | phosphodiesterase 1C | 9 | 1 | 0.0273 | 0.0227 | 0.011 |
| PDE1B | cam-PDE 1B | 9 | 1 | 0.0038 | 0.0056 | 0.0042 |
| CHRM3 | cholinergic receptor muscarinic 3 | 9 | 2 | 0.0164 | 0.0117 | 0.006 |
| WNT7A | Wnt family member 7A | 9 | 1 | 0.0013 | 0.0084 | 0.0015 |
| FZD7 | frizzled class receptor 7 | 9 | 1 | 0.0046 | 0.0074 | 0.0025 |
| TLN2 | talin 2 | 9 | 2 | 0.003 | 0.0078 | 0.0031 |
| GRIN2A | glutamate ionotropic receptor NMDA type subunit 2A | 9 | 6 | 0.0054 | 0.0048 | 0.0025 |
| VAV3 | vav guanine nucleotide exchange factor 3 | 9 | 1 | 0.0033 | 0.005 | 0.0104 |
| VAV2 | vav guanine nucleotide exchange factor 2 | 9 | 1 | 0.0166 | 0.0086 | 0.0074 |
| PPP3CA | protein phosphatase 3 catalytic subunit alpha | 9 | 7 | 0.0124 | 0.0101 | 0.0065 |
| CAMK4 | calcium/calmodulin dependent protein kinase IV | 8 | 2 | 0.0037 | 0.0114 | 0.0042 |
| SCTR | secretin receptor | 8 | 1 | 0.0012 | 0.0034 | 0.003 |
| GUCY1B3 | guanylate cyclase 1 soluble subunit beta | 8 | 4 | 0.0033 | 0.0033 | 0.0011 |
| GUCY1A2 | guanylate cyclase 1 soluble subunit alpha 2 | 8 | 4 | 0.0232 | 0.0105 | 0.018 |
| GRM7 | glutamate metabotropic receptor 7 | 8 | 2 | 0.0138 | 0.0334 | 0.0102 |
| GRM3 | glutamate metabotropic receptor 3 | 8 | 2 | 0.0158 | 0.0152 | 0.0035 |
| HTR7 | 5-hydroxytryptamine receptor 7 | 8 | 2 | 0.0076 | 0.0016 | 0.0019 |
| CACNA1B | calcium voltage-gated channel subunit alpha1 B | 8 | 4 | 0.0062 | 0.0034 | 0.0135 |
| MAP3K5 | mitogen-activated protein kinase kinase kinase 5 | 8 | 2 | 0.008 | 0.0148 | 0.0172 |

| Gene name | Gene description | Number of Connections (n) | Number of pathways (n) | Genetic variance (%) | | |
|---|---|---|---|---|---|---|
| | | | | MY | FY | AFC |
| PPAP2B | phosphatidic acid phosphatase type 2B | 8 | 1 | 0.0055 | 0.0027 | 0.0018 |
| CREBBP | CREB binding protein | 8 | 1 | 0.0049 | 0.0081 | 0.0099 |
| BTRC | beta-transducin repeat containing E3 ubiquitin protein ligase | 8 | 1 | 0.004 | 0.0136 | 0.0049 |
| ITGAL | integrin subunit alpha L | 8 | 1 | 0.0025 | 0.0044 | 0.0018 |
| LCP2 | lymphocyte cytosolic protein 2\|tyrosine phosphoprotein slp-76 | 8 | 1 | 0.0054 | 0.004 | 0.0104 |
| CACNA2D1 | calcium voltage-gated channel auxiliary subunit alpha2delta 1 | 7 | 2 | 0.0104 | 0.0071 | 0.0022 |
| LAMA5 | laminin subunit alpha 5 | 7 | 1 | 0.0035 | 0.0042 | 0.0045 |
| CACNA1E | calcium voltage-gated channel subunit alpha1 E | 7 | 2 | 0.0146 | 0.0107 | 0.0044 |
| COL9A3 | collagen type IX alpha 3 chain | 7 | 1 | 0.0052 | 0.005 | 0.0105 |
| COL2A1 | collagen type II alpha 1 chain | 7 | 1 | 0.0046 | 0.0026 | 0.0016 |
| GABBR2 | gamma-aminobutyric acid type B receptor subunit 2 | 7 | 1 | 0.0099 | 0.0063 | 0.0301 |
| ITPR2 | inositol 1,4,5-triphosphate receptor, type 2 | 7 | 7 | 0.0071 | 0.0083 | 0.0192 |
| HTR4 | 5-hydroxytryptamine receptor 4 | 7 | 1 | 0.0098 | 0.0149 | 0.0107 |
| COL4A5 | collagen type IV alpha 5 chain | 7 | 1 | 0.0074 | 0.0037 | 0.0106 |
| ITPR1 | IP3 receptor | 7 | 8 | 0.0126 | 0.0084 | 0.0057 |
| PTK2B | protein tyrosine kinase 2 beta | 7 | 2 | 0.0015 | 0.0013 | 0.0047 |
| LAMC2 | laminin subunit gamma 2 | 7 | 1 | 0.0053 | 0.0044 | 0.015 |
| AGPAT4 | lysophosphatidic acid acyltransferase delta | 7 | 1 | 0.0141 | 0.0228 | 0.0021 |
| LAMA3 | laminin subunit alpha 3 | 7 | 1 | 0.0041 | 0.0014 | 0.0047 |
| MAP2K6 | mitogen-activated protein kinase kinase 6 | 7 | 2 | 0.0019 | 0.0051 | 0.0066 |
| WNT11 | Wnt family member 11 | 7 | 1 | 0.0013 | 0.0029 | 0.0024 |
| CHUK | conserved helix-loop-helix ubiquitous kinase | 7 | 2 | 0.0017 | 0.0095 | 0.0077 |
| ERBB4 | erb-b2 receptor tyrosine kinase 4 | 7 | 1 | 0.0229 | 0.014 | 0.0271 |
| PPP3CC | protein phosphatase 3 catalytic subunit gamma | 7 | 7 | 0.0079 | 0.0028 | 0.0045 |

| Gene name | Gene description | Number of Connections (n) | Number of pathways (n) | Genetic variance (%) | | |
|---|---|---|---|---|---|---|
| | | | | MY | FY | AFC |
| PLCL1 | phospholipase C like 1 | 6 | 1 | 0.0115 | 0.014 | 0.0129 |
| RYR2 | ryanodine receptor 2 | 6 | 4 | 0.0083 | 0.0245 | 0.0183 |
| GABRB1 | gamma-aminobutyric acid type A receptor beta1 subunit | 6 | 1 | 0.0158 | 0.0071 | 0.0046 |
| PTGER3 | prostaglandin E receptor 3 | 6 | 1 | 0.002 | 0.0027 | 0.0026 |
| TIAM1 | T-cell lymphoma invasion and metastasis 1 | 6 | 2 | 0.0114 | 0.0043 | 0.0189 |
| COL4A2 | collagen type IV alpha 2 chain | 6 | 1 | 0.004 | 0.0037 | 0.0064 |
| COL4A1 | collagen type IV alpha 1 chain | 6 | 1 | 0.0075 | 0.004 | 0.0109 |
| WNT10B | Wnt family member 10B | 6 | 1 | 0.0036 | 0.0025 | 0.002 |
| ABL1 | ABL proto-oncogene 1, non-receptor tyrosine kinase | 6 | 1 | 0.0066 | 0.0054 | 0.0049 |
| WIF1 | WNT inhibitory factor 1 | 6 | 1 | 0.0013 | 0.0028 | 0.0068 |
| PIP5K1B | phosphatidylinositol-4-phosphate 5-kinase type 1 beta | 6 | 1 | 0.0166 | 0.0115 | 0.0071 |
| WNT9B | Wnt family member 9B | 6 | 1 | 0.0032 | 0.0012 | 0.0047 |
| LEF1 | lymphoid enhancer binding factor 1 | 6 | 1 | 0.0128 | 0.0067 | 0.0016 |
| TAB2 | TGF-beta activated kinase 1/MAP3K7 binding protein 2 | 6 | 1 | 0.0126 | 0.0096 | 0.0132 |
| MAP2K4 | mitogen-activated protein kinase kinase 4 | 6 | 1 | 0.0016 | 0.0059 | 0.0018 |
| APC | APC, WNT signaling pathway regulator | 6 | 1 | 0.0051 | 0.0136 | 0.0054 |
| TSC2 | tuberous sclerosis 2 | 6 | 1 | 0.0053 | 0.0032 | 0.0028 |
| CHAD | chondroadherin | 5 | 1 | 0.0018 | 0.0017 | 0.0016 |
| CD38 | CD38 molecule | 5 | 3 | 0.004 | 0.0056 | 0.0077 |
| DGKH | diacylglycerol kinase eta | 5 | 1 | 0.0168 | 0.0149 | 0.004 |
| LAMA4 | laminin subunit alpha 4 | 5 | 1 | 0.0118 | 0.0095 | 0.0027 |
| TNR | tenascin R | 5 | 1 | 0.0144 | 0.0241 | 0.0053 |
| GABRA5 | gamma-aminobutyric acid type A receptor alpha5 subunit | 5 | 1 | 0.0195 | 0.0109 | 0.0051 |

| Gene name | Gene description | Number of Connections (n) | Number of pathways (n) | Genetic variance (%) | | |
|---|---|---|---|---|---|---|
| | | | | MY | FY | AFC |
| GABRB2 | gamma-aminobutyric acid type A receptor beta2 subunit | 5 | 1 | 0.0036 | 0.0053 | 0.0147 |
| DGKE | diacylglycerol kinase epsilon | 5 | 1 | 0.0024 | 0.0018 | 0.0017 |
| THBS2 | thrombospondin 2 | 5 | 1 | 0.011 | 0.0046 | 0.0016 |
| PARVA | parvin alpha | 5 | 1 | 0.0074 | 0.0092 | 0.0152 |
| DGKG | diacylglycerol kinase gamma | 5 | 1 | 0.0025 | 0.0019 | 0.0024 |
| DGKB | diacylglycerol kinase beta | 5 | 1 | 0.0205 | 0.0188 | 0.01 |
| DGKI | diacylglycerol kinase iota | 5 | 1 | 0.0338 | 0.0218 | 0.0199 |
| SGMS2 | sphingomyelin synthase 2 | 5 | 1 | 0.0011 | 0.0016 | 0.0035 |
| GPHN | gephyrin | 5 | 1 | 0.0337 | 0.0289 | 0.0188 |
| SGMS1 | sphingomyelin synthase 1 | 5 | 1 | 0.0012 | 0.0022 | 0.0058 |
| CERS6 | ceramide synthase 6 | 5 | 1 | 0.0096 | 0.0012 | 0.0095 |
| DEGS1 | delta 4-desaturase, sphingolipid 1 | 5 | 1 | 0.0027 | 0.0043 | 0.0117 |
| CERS3 | ceramide synthase 3 | 5 | 1 | 0.0047 | 0.0045 | 0.0024 |
| TNC | tenascin C | 5 | 1 | 0.0032 | 0.0042 | 0.0084 |
| ARRB1 | arrestin beta 1 | 5 | 1 | 0.0066 | 0.0095 | 0.006 |
| TCF7L2 | transcription factor 7 like 2 | 5 | 1 | 0.0133 | 0.0098 | 0.0117 |
| PPP3R1 | protein phosphatase 3 regulatory subunit B, alpha | 5 | 6 | 0.0012 | 0.0032 | 0.0011 |
| KIF5A | kinesin family member 5A | 4 | 1 | 0.0098 | 0.0216 | 0.009 |
| KIF5C | kinesin family member 5C | 4 | 1 | 0.002 | 0.0017 | 0.0016 |
| MAP2K5 | mitogen-activated protein kinase kinase 5 | 4 | 3 | 0.0147 | 0.0184 | 0.0063 |
| MAGI3 | membrane associated guanylate kinase, WW and PDZ domain containing 3 | 4 | 1 | 0.0065 | 0.0047 | 0.0017 |
| RAPGEF6 | Rap guanine nucleotide exchange factor 6 | 4 | 1 | 0.0052 | 0.0052 | 0.0094 |
| RAPGEF2 | Rap guanine nucleotide exchange factor 2 | 4 | 2 | 0.009 | 0.0112 | 0.0019 |
| GRIK3 | Glutamate Ionotropic Receptor Kainate Type Subunit 3 | 4 | 1 | 0.0063 | 0.0061 | 0.0115 |

| Gene name | Gene description | Number of Connections (n) | Number of pathways (n) | Genetic variance (%) | | |
|---|---|---|---|---|---|---|
| | | | | MY | FY | AFC |
| LPAR1 | lysophosphatidic acid receptor 1 | 4 | 3 | 0.0155 | 0.0037 | 0.0057 |
| NTRK2 | neurotrophic receptor tyrosine kinase 2 | 4 | 1 | 0.0095 | 0.0058 | 0.0011 |
| FBXW11 | F-box and WD repeat domain containing 11 | 4 | 1 | 0.0032 | 0.0012 | 0.0089 |
| PLA2G16 | phospholipase A2, group XVI | 4 | 1 | 0.0019 | 0.0029 | 0.0018 |
| GABRG2 | Gamma-Aminobutyric Acid Type A Receptor Gamma2 Subunit | 4 | 1 | 0.0041 | 0.0063 | 0.0142 |
| NFATC2 | nuclear factor of activated T-cells 2 | 4 | 3 | 0.0138 | 0.0106 | 0.0087 |
| RPS6KA2 | ribosomal protein S6 kinase A2 | 4 | 1 | 0.0056 | 0.01 | 0.0134 |
| RPS6KA3 | ribosomal protein S6 kinase A3 | 4 | 1 | 0.0039 | 0.0023 | 0.005 |
| MAPKAPK2 | mitogen-activated protein kinase-activated protein kinase 2 | 4 | 1 | 0.0051 | 0.0031 | 0.0057 |
| MRVI1 | murine retrovirus integration site 1 homolog | 4 | 1 | 0.0049 | 0.0103 | 0.0171 |
| SLC8A3 | solute carrier family 8 member A3 | 3 | 2 | 0.0142 | 0.0106 | 0.0023 |
| SLC8A1 | solute carrier family 8 member A1 | 3 | 2 | 0.0126 | 0.0137 | 0.0265 |
| PRKD1 | protein kinase D1 | 3 | 1 | 0.0077 | 0.0091 | 0.0181 |
| MAGI1 | membrane associated guanylate kinase, WW and PDZ domain containing 1 | 3 | 1 | 0.0131 | 0.0151 | 0.0043 |
| STK3 | serine/threonine kinase 3 | 3 | 1 | 0.0141 | 0.0127 | 0.0102 |
| DKK4 | dickkopf WNT signaling pathway inhibitor 4 | 3 | 1 | 0.0044 | 0.0026 | 0.0054 |
| BID | BH3 Interacting Domain Death Agonist | 3 | 1 | 0.0012 | 0.0031 | 0.003 |
| CAV2 | caveolin 2 | 3 | 1 | 0.0029 | 0.0019 | 0.001 |
| ADRA1A | adrenoceptor alpha 1A | 3 | 2 | 0.006 | 0.0024 | 0.0084 |
| SGPP1 | sphingosine-1-phosphate phosphatase 1 | 3 | 1 | 0.0013 | 0.0049 | 0.003 |
| ITPKB | inositol-trisphosphate 3-kinase B | 3 | 1 | 0.0068 | 0.0044 | 0.0054 |
| PLA2G2F | phospholipase A2 group IIF | 3 | 2 | 0.0025 | 0.0018 | 0.0026 |
| GABRA3 | gamma-aminobutyric acid type A receptor alpha3 subunit | 3 | 1 | 0.0032 | 0.0021 | 0.0031 |

| Gene name | Gene description | Number of Connections (n) | Number of pathways (n) | Genetic variance (%) | | |
|---|---|---|---|---|---|---|
| | | | | MY | FY | AFC |
| FRAT2 | FRAT2, WNT signaling pathway regulator | 3 | 1 | 0.0017 | 0.0018 | 0.0016 |
| SKAP1 | src kinase associated phosphoprotein 1 | 3 | 1 | 0.0108 | 0.0077 | 0.0072 |
| DKK2 | dickkopf WNT signaling pathway inhibitor 2 | 3 | 1 | 0.008 | 0.0063 | 0.0047 |
| KCNMB2 | potassium calcium-activated channel subfamily M regulatory beta subunit 2 | 3 | 1 | 0.0055 | 0.0046 | 0.0056 |
| KCNMA1 | potassium calcium-activated channel subfamily M alpha 1 | 3 | 2 | 0.033 | 0.0273 | 0.0221 |
| PRKCE | protein kinase C epsilon | 3 | 2 | 0.0345 | 0.0509 | 0.0184 |
| PLA2G12B | phospholipase A2 group XIIB | 3 | 2 | 0.001 | 0.0014 | 0.01 |
| PTPRR | protein tyrosine phosphatase, receptor type R | 3 | 1 | 0.0187 | 0.0024 | 0.0112 |
| RPS6KA5 | ribosomal protein S6 kinase A5 | 3 | 2 | 0.0067 | 0.0082 | 0.007 |
| DUSP10 | dual specificity phosphatase 10 | 3 | 1 | 0.0302 | 0.0381 | 0.0175 |
| NLK | nemo like kinase | 3 | 2 | 0.0081 | 0.0111 | 0.0052 |
| FYB | FYN binding protein | 3 | 1 | 0.0049 | 0.0013 | 0.0075 |
| MKNK1 | MAP Kinase Interacting Serine/Threonine Kinase 1 | 2 | 1 | 0.0022 | 0.0118 | 0.006 |
| ETS1 | ETS proto-oncogene 1, transcription factor | 2 | 1 | 0.0062 | 0.0124 | 0.0022 |
| ATP2B2 | ATPase plasma membrane Ca2+ transporting 2 | 2 | 3 | 0.006 | 0.0104 | 0.0082 |
| ATP2B4 | ATPase plasma membrane Ca2+ transporting 4 | 2 | 3 | 0.0025 | 0.0034 | 0.0082 |
| DNM2 | dynamin 2 | 2 | 1 | 0.0027 | 0.0024 | 0.0035 |
| STK4 | serine/threonine kinase 4 | 2 | 2 | 0.0084 | 0.004 | 0.0071 |
| RBX1 | Ring-Box 1 | 2 | 1 | 0.0038 | 0.0017 | 0.0016 |
| PPP1R12A | protein phosphatase 1 regulatory subunit 12A | 2 | 3 | 0.0037 | 0.0023 | 0.0013 |
| PIK3R6 | phosphoinositide-3-kinase regulatory subunit 6 | 2 | 3 | 0.0014 | 0.0027 | 0.0014 |
| CTSD | cathepsin D | 2 | 1 | 0.0028 | 0.0011 | 0.0022 |
| TRPC6 | transient receptor potential cation channel subfamily C member 6 | 2 | 1 | 0.0227 | 0.0424 | 0.0034 |
| LOC529488 | glutamate decarboxylase 1 | 2 | 1 | 0.0054 | 0.0038 | 0.016 |

| Gene name | Gene description | Number of Connections (n) | Number of pathways (n) | Genetic variance (%) | | |
|---|---|---|---|---|---|---|
| | | | | MY | FY | AFC |
| GLS | glutaminase | 2 | 2 | 0.0029 | 0.0032 | 0.0015 |
| GAD2 | glutamate decarboxylase 2 | 2 | 1 | 0.0045 | 0.0035 | 0.0018 |
| FCER1A | Fc fragment of IgE receptor Ia | 2 | 2 | 0.0037 | 0.0087 | 0.0045 |
| MS4A2 | Membrane Spanning 4-Domains A2 | 2 | 2 | 0.0031 | 0.0025 | 0.0029 |
| ARF1 | ADP ribosylation factor 1 | 2 | 1 | 0.0029 | 0.0012 | 0.0021 |
| SHANK2 | SH3 and multiple ankyrin repeat domains 2 | 2 | 1 | 0.0192 | 0.0098 | 0.0255 |
| DLGAP1 | DLG associated protein 1 | 2 | 1 | 0.0041 | 0.0099 | 0.0028 |
| SHANK1 | SH3 and multiple ankyrin repeat domains 1 | 2 | 1 | 0.0054 | 0.0059 | 0.0012 |
| TCF7L1 | transcription factor 7 like 1 | 2 | 1 | 0.0252 | 0.017 | 0.002 |
| ADRBK2 | G Protein-Coupled Receptor Kinase 2 | 2 | 1 | 0.0013 | 0.0012 | 0.0098 |
| PPM1B | protein phosphatase, Mg2+/Mn2+ dependent 1B | 1 | 1 | 0.0017 | 0.0013 | 0.0028 |
| CFTR | ATP-binding cassette sub-family C member 7 | 1 | 1 | 0.0149 | 0.009 | 0.0101 |
| DNM3 | dynamin 3 | 1 | 1 | 0.0083 | 0.0039 | 0.0037 |
| ARHGAP35 | Rho GTPase activating protein 35 | 1 | 1 | 0.0011 | 0.01 | 0.0031 |
| IL1R1 | interleukin 1 receptor type 1 | 1 | 1 | 0.0128 | 0.0086 | 0.0021 |
| SLC17A8 | solute carrier family 17 member 8 | 1 | 1 | 0.0026 | 0.0042 | 0.0056 |
| SLC18A2 | solute carrier family 18 member A2 | 1 | 1 | 0.0021 | 0.0016 | 0.0042 |
| MECOM | MDS1 and EVI1 complex locus | 1 | 1 | 0.0167 | 0.0178 | 0.0216 |
| KSR2 | kinase suppressor of ras 2 | 1 | 1 | 0.0045 | 0.0102 | 0.0094 |
| MYLK3 | myosin light chain kinase 3 | 1 | 4 | 0.0031 | 0.0063 | 0.0015 |
| PAK7 | P21 (RAC1) Activated Kinase 5 | 1 | 2 | 0.0076 | 0.0026 | 0.0079 |
| PARD3 | par-3 family cell polarity regulator | 1 | 1 | 0.0014 | 0.0042 | 0.0175 |
| DOCK4 | dedicator of cytokinesis 4 | 1 | 1 | 0.011 | 0.0168 | 0.004 |
| DOCK1 | dedicator of cytokinesis 1 | 1 | 1 | 0.0428 | 0.0386 | 0.027 |
| TGFB2 | transforming growth factor beta 2 | 1 | 1 | 0.0021 | 0.0023 | 0.01 |
| MYL12B | myosin, light chain 12B, regulatory | 1 | 1 | 0.0049 | 0.0017 | 0.0103 |
| P2RX1 | purinergic receptor P2X 1 | 1 | 1 | 0.0012 | 0.0011 | 0.0034 |

| Gene name | Gene description | Number of Connections (n) | Number of pathways (n) | Genetic variance (%) | | |
|---|---|---|---|---|---|---|
| | | | | MY | FY | AFC |
| P2RX3 | purinergic receptor P2X 3 | 1 | 1 | 0.0049 | 0.0015 | 0.0012 |
| SIPA1L1 | signal induced proliferation associated 1 like 1 | 1 | 1 | 0.0196 | 0.0343 | 0.0496 |
| GRIK4 | glutamate ionotropic receptor kainate type subunit 4 | 1 | 1 | 0.0101 | 0.0073 | 0.0205 |
| MAPT | PHF-tau\|microtubule associated protein tau\|neurofibrillary tangle protein\|paired helical filament-tau | 1 | 1 | 0.002 | 0.0013 | 0.0012 |
| PHKB | phosphorylase kinase regulatory subunit beta | 1 | 1 | 0.0222 | 0.0207 | 0.0049 |
| PHKA2 | Phosphorylase Kinase Regulatory Subunit Alpha 2 | 1 | 1 | 0.0034 | 0.0035 | 0.0019 |
| RAP1GAP | RAP1 GTPase activating protein | 1 | 1 | 0.0011 | 0.0027 | 0.0016 |
| **Total** | | **2,313** | **625** | **2.2789** | **2.2624** | **2.1223** |

**Supplementary File S1** *Number of connections, number of pathways and predicted SNP value for milk yield (MY), fat yield (FY), and age at first calving (AFC) explained by single nucleotide polymorphism located inside or within 2,500 bp of genes in the Thai multibreed population.*

| Gene name | Gene description | Number of Connections (n) | Number of pathways (n) | Predicted SNP value | | |
|---|---|---|---|---|---|---|
| | | | | MY | FY | AFC |
| PLCB1 | phospholipase C beta 1 | 44 | 12 | -0.2944 | -0.0171 | -0.0001 |
| GNG2 | G protein subunit gamma 2 | 38 | 5 | -0.0437 | 0.0008 | -0.0009 |
| PLCB4 | phosphoinositide phospholipase C | 34 | 12 | -0.1088 | -0.0040 | 0.0024 |
| CAMK2B | calcium/calmodulin dependent protein kinase II beta | 32 | 5 | 0.0128 | -0.0021 | -0.0001 |
| CAMK2D | calcium/calmodulin dependent protein kinase II delta | 32 | 5 | -0.1764 | 0.0042 | 0.0004 |
| MAPK14 | mitogen-activated protein kinase 14 | 29 | 4 | 0.1379 | 0.0040 | -0.0007 |
| GRB2 | growth factor receptor bound protein 2 | 29 | 5 | 0.0972 | 0.0030 | 0.0000 |
| ITGB5 | integrin subunit beta 5 | 27 | 1 | -0.0167 | -0.0029 | -0.0003 |
| PRKCB | protein kinase C beta | 25 | 14 | 0.2256 | 0.0051 | -0.0015 |
| FYN | FYN proto-oncogene, Src family tyrosine kinase | 24 | 3 | -0.1183 | -0.0044 | -0.0005 |
| GNGT1 | G protein subunit gamma transducin 1 | 22 | 5 | -0.1447 | -0.0039 | -0.0004 |
| ADCY8 | adenylate cyclase 8 | 22 | 10 | -0.4131 | -0.0050 | 0.0001 |
| ADCY2 | adenylate cyclase 2 | 22 | 10 | 0.0889 | -0.0008 | 0.0003 |
| GNAO1 | G protein subunit alpha o1 | 22 | 6 | 0.0117 | 0.0043 | -0.0011 |
| MAPK11 | mitogen-activated protein kinase 11 | 22 | 4 | 0.0898 | 0.0029 | 0.0004 |
| EGFR | epidermal growth factor receptor | 22 | 8 | -0.0384 | -0.0041 | -0.0008 |
| ADCY5 | adenylate cyclase 5 | 21 | 10 | 0.1311 | 0.0017 | 0.0003 |
| ADCY7 | adenylate cyclase 7 | 21 | 10 | -0.2207 | -0.0109 | -0.0004 |

| Gene name | Gene description | Number of Connections (n) | Number of pathways (n) | Predicted SNP value | | |
|---|---|---|---|---|---|---|
| | | | | MY | FY | AFC |
| GNAQ | G protein subunit alpha q | 21 | 10 | -0.0211 | -0.0060 | 0.0005 |
| RAP1B | RAP1B, member of RAS oncogene family | 21 | 5 | 0.1595 | 0.0079 | -0.0005 |
| IRS1 | insulin receptor substrate 1 | 21 | 1 | -0.2775 | -0.0046 | 0.0005 |
| PRKCA | protein kinase C alpha | 20 | 15 | -0.0481 | 0.0059 | -0.0002 |
| ITGA6 | integrin subunit alpha 6 | 20 | 1 | 0.2281 | 0.0035 | -0.0004 |
| ITGA9 | integrin subunit alpha 9 | 20 | 1 | -0.0938 | 0.0089 | -0.0013 |
| ITGA8 | integrin subunit alpha 8 | 20 | 1 | -0.5823 | -0.0127 | 0.0020 |
| PTEN | phosphatase and tensin homolog | 20 | 2 | -0.1400 | -0.0048 | 0.0004 |
| AKT3 | AKT serine/threonine kinase 3 | 20 | 8 | -0.0024 | 0.0022 | 0.0010 |
| ITGA2 | integrin subunit alpha 2 | 19 | 1 | 0.0841 | 0.0023 | 0.0011 |
| IGF1R | insulin like growth factor 1 receptor | 19 | 3 | -0.1536 | -0.0035 | -0.0003 |
| RASGRF2 | RAS protein-specific guanine nucleotide-releasing factor 2 | 18 | 2 | -0.2245 | -0.0025 | 0.0001 |
| PPP2R5E | protein phosphatase 2 regulatory subunit B'epsilon | 18 | 2 | 0.0000 | 0.0000 | 0.0000 |
| PDGFRA | platelet derived growth factor receptor alpha | 18 | 7 | -0.0728 | 0.0026 | -0.0004 |
| PDGFRB | platelet derived growth factor receptor beta | 18 | 7 | -0.1023 | -0.0048 | 0.0011 |
| MAPK10 | mitogen-activated protein kinase 10 | 18 | 6 | -0.2962 | -0.0055 | 0.0018 |
| CACNG2 | calcium voltage-gated channel auxiliary subunit gamma 2 | 18 | 2 | 0.2016 | 0.0034 | -0.0009 |
| SOS1 | SOS Ras/Rac guanine nucleotide exchange factor 1 | 18 | 5 | -0.1791 | -0.0039 | 0.0007 |
| PRKG2 | protein kinase, cGMP-dependent, type II | 17 | 3 | -0.0887 | -0.0033 | 0.0000 |
| PRKG1 | protein kinase, cGMP-dependent, type I | 17 | 3 | 0.1390 | -0.0035 | 0.0008 |
| ADCY1 | adenylate cyclase 1 | 16 | 10 | -0.0846 | -0.0026 | 0.0004 |
| LPAR3 | lysophosphatidic acid receptor 3 | 16 | 2 | 0.0617 | 0.0010 | -0.0001 |
| GRM1 | glutamate receptor, metabotropic 1 | 16 | 4 | 0.1673 | 0.0094 | -0.0006 |
| PLCE1 | phospholipase C epsilon 1 | 16 | 3 | 0.1183 | 0.0093 | 0.0010 |
| INSR | insulin receptor | 16 | 4 | -0.3107 | -0.0131 | 0.0012 |

| Gene name | Gene description | Number of Connections (n) | Number of pathways (n) | Predicted SNP value | | |
|---|---|---|---|---|---|---|
| | | | | MY | FY | AFC |
| SHC3 | SHC adaptor protein 3 | 16 | 3 | 0.0664 | -0.0008 | 0.0001 |
| CACNG4 | calcium voltage-gated channel auxiliary subunit gamma 4 | 15 | 2 | -0.0842 | -0.0021 | 0.0006 |
| GSK3B | glycogen synthase kinase 3 beta | 15 | 3 | -0.3226 | -0.0041 | 0.0003 |
| PPP2R2B | protein phosphatase 2 regulatory subunit Bbeta | 14 | 2 | 0.0603 | 0.0021 | 0.0004 |
| CACNB2 | calcium voltage-gated channel auxiliary subunit beta 2 | 14 | 2 | -0.0464 | 0.0024 | -0.0005 |
| FLT1 | fms related tyrosine kinase 1 | 14 | 3 | -0.1673 | -0.0024 | -0.0003 |
| GRIA3 | glutamate ionotropic receptor AMPA type subunit 3 | 14 | 3 | 0.2136 | 0.0063 | -0.0012 |
| MET | MET proto-oncogene, receptor tyrosine kinase | 14 | 3 | -0.1268 | -0.0027 | -0.0005 |
| GRIA1 | glutamate receptor, ionotropic, AMPA1 | 14 | 3 | -0.1594 | -0.0024 | 0.0008 |
| TEK | TEK receptor tyrosine kinase | 14 | 2 | -0.0937 | -0.0014 | -0.0003 |
| RASGRP3 | RAS guanyl releasing protein 3 | 13 | 3 | 0.1886 | 0.0046 | -0.0004 |
| PLD1 | phospholipase D1, phosphatidylcholine-specific | 13 | 4 | 0.1959 | 0.0023 | -0.0007 |
| FGF19 | fibroblast growth factor 19 | 13 | 3 | 0.1805 | 0.0027 | -0.0004 |
| EFNA5 | ephrin A5 | 13 | 2 | -0.0419 | 0.0000 | 0.0004 |
| CACNB4 | calcium voltage-gated channel auxiliary subunit beta 4 | 13 | 2 | 0.2408 | -0.0035 | 0.0001 |
| GNA12 | G protein subunit alpha 12 | 13 | 4 | 0.2226 | 0.0025 | 0.0002 |
| FGF2 | BFGF | 13 | 3 | -0.0823 | -0.0019 | 0.0003 |
| TLN1 | talin 1 | 13 | 2 | -0.0808 | -0.0029 | -0.0004 |
| GRIA2 | glutamate ionotropic receptor AMPA type subunit 2 | 13 | 3 | -0.0639 | -0.0021 | -0.0006 |
| FLT4 | fms related tyrosine kinase 4 | 13 | 3 | 0.0666 | 0.0018 | -0.0007 |
| PDE3B | phosphodiesterase 3B | 12 | 1 | -0.2859 | -0.0064 | 0.0003 |
| EDNRB | endothelin receptor type B | 12 | 2 | -0.0924 | -0.0021 | 0.0008 |
| CACNA1A | calcium voltage-gated channel subunit alpha1 A | 12 | 5 | -0.7185 | -0.0176 | 0.0015 |

| Gene name | Gene description | Number of Connections (n) | Number of pathways (n) | Predicted SNP value | | |
|---|---|---|---|---|---|---|
| | | | | MY | FY | AFC |
| GNA13 | G protein subunit alpha 13 | 12 | 3 | 0.1230 | 0.0040 | -0.0004 |
| GRIA4 | glutamate ionotropic receptor AMPA type subunit 4 | 12 | 3 | 0.1124 | -0.0015 | -0.0010 |
| ITGB2 | integrin subunit beta 2 | 12 | 1 | -0.1126 | -0.0033 | 0.0005 |
| ANGPT1 | angiopoietin 1 | 12 | 2 | 0.0392 | 0.0000 | 0.0006 |
| PDE3A | phosphodiesterase 3A | 11 | 1 | -0.1269 | -0.0038 | 0.0008 |
| CACNA2D4 | calcium voltage-gated channel auxiliary subunit alpha2delta 4 | 11 | 2 | 0.0682 | 0.0031 | 0.0003 |
| CACNA2D3 | calcium voltage-gated channel auxiliary subunit alpha2delta 3 | 11 | 2 | -0.0887 | -0.0096 | -0.0015 |
| FN1 | fibronectin 1 | 11 | 1 | 0.0863 | 0.0048 | -0.0003 |
| BCL2 | BCL2, apoptosis regulator | 11 | 2 | 0.0541 | -0.0008 | 0.0003 |
| GRIN2B | glutamate ionotropic receptor NMDA type subunit 2B | 11 | 5 | 0.1044 | -0.0006 | 0.0002 |
| GRIN1 | glutamate ionotropic receptor NMDA type subunit 1 | 11 | 5 | 0.0835 | 0.0033 | -0.0006 |
| LRP6 | LDL receptor related protein 6 | 11 | 1 | -0.0717 | -0.0014 | -0.0004 |
| CACNG5 | calcium voltage-gated channel auxiliary subunit gamma 5 | 10 | 2 | 0.0748 | 0.0047 | 0.0003 |
| FGF12 | fibroblast growth factor 12 | 10 | 3 | -0.1499 | -0.0133 | 0.0010 |
| PDGFD | platelet derived growth factor D | 10 | 5 | -0.2855 | -0.0100 | -0.0001 |
| PDGFC | platelet derived growth factor C | 10 | 5 | -0.3096 | -0.0043 | 0.0003 |
| CASP3 | caspase 3, apoptosis-related cysteine peptidase | 10 | 1 | -0.0839 | -0.0029 | 0.0003 |
| RAPGEF4 | Rap guanine nucleotide exchange factor 4 | 10 | 2 | -0.1286 | 0.0001 | -0.0007 |
| FLNB | filamin B | 10 | 2 | -0.2384 | -0.0063 | -0.0001 |
| LRP5 | LDL receptor related protein 5 | 10 | 1 | -0.0695 | 0.0016 | 0.0003 |
| FIGF | c-fos induced growth factor | 10 | 3 | -0.1949 | -0.0059 | 0.0005 |
| WNT3A | Wnt family member 3A | 10 | 1 | 0.1754 | 0.0025 | -0.0004 |

| Gene name | Gene description | Number of Connections (n) | Number of pathways (n) | Predicted SNP value | | |
|---|---|---|---|---|---|---|
| | | | | MY | FY | AFC |
| GAB1 | GRB2 associated binding protein 1 | 10 | 2 | -0.1477 | -0.0071 | 0.0003 |
| TACR1 | tachykinin receptor 1 | 9 | 1 | -0.2235 | -0.0053 | -0.0001 |
| TACR3 | tachykinin receptor 3 | 9 | 1 | 0.0760 | 0.0056 | -0.0008 |
| NTSR1 | neurotensin receptor 1 | 9 | 1 | -0.0747 | -0.0026 | -0.0004 |
| NSF | N-ethylmaleimide sensitive factor, vesicle fusing ATPase | 9 | 1 | -0.0277 | -0.0005 | 0.0005 |
| PDE1A | phosphodiesterase 1A, calmodulin-dependent | 9 | 1 | 0.2022 | 0.0020 | 0.0003 |
| PDE1C | phosphodiesterase 1C | 9 | 1 | 0.2922 | 0.0061 | -0.0007 |
| PDE1B | cam-PDE 1B | 9 | 1 | -0.1092 | -0.0037 | 0.0005 |
| CHRM3 | cholinergic receptor muscarinic 3 | 9 | 2 | -0.3344 | -0.0051 | 0.0008 |
| WNT7A | Wnt family member 7A | 9 | 1 | 0.0411 | 0.0018 | 0.0004 |
| FZD7 | frizzled class receptor 7 | 9 | 1 | -0.1394 | -0.0049 | -0.0004 |
| TLN2 | talin 2 | 9 | 2 | -0.2003 | -0.0057 | -0.0005 |
| GRIN2A | glutamate ionotropic receptor NMDA type subunit 2A | 9 | 6 | -0.0682 | -0.0011 | 0.0000 |
| VAV3 | vav guanine nucleotide exchange factor 3 | 9 | 1 | -0.1188 | -0.0008 | 0.0001 |
| VAV2 | vav guanine nucleotide exchange factor 2 | 9 | 1 | -0.1439 | -0.0036 | -0.0009 |
| PPP3CA | protein phosphatase 3 catalytic subunit alpha | 9 | 7 | -0.3663 | -0.0125 | 0.0011 |
| CAMK4 | calcium/calmodulin dependent protein kinase IV | 8 | 2 | 0.0132 | 0.0028 | -0.0006 |
| SCTR | secretin receptor | 8 | 1 | 0.0417 | 0.0044 | 0.0000 |
| GUCY1B3 | guanylate cyclase 1 soluble subunit beta | 8 | 4 | -0.2392 | -0.0053 | -0.0003 |
| GUCY1A2 | guanylate cyclase 1 soluble subunit alpha 2 | 8 | 4 | 0.3172 | 0.0096 | -0.0011 |
| GRM7 | glutamate metabotropic receptor 7 | 8 | 2 | -0.1710 | -0.0019 | -0.0012 |
| GRM3 | glutamate metabotropic receptor 3 | 8 | 2 | -0.0206 | -0.0048 | -0.0007 |
| HTR7 | 5-hydroxytryptamine receptor 7 | 8 | 2 | -0.2181 | 0.0021 | -0.0003 |
| CACNA1B | calcium voltage-gated channel subunit alpha1 B | 8 | 4 | -0.0744 | 0.0040 | -0.0005 |
| MAP3K5 | mitogen-activated protein kinase kinase kinase 5 | 8 | 2 | 0.1774 | 0.0117 | -0.0011 |

| Gene name | Gene description | Number of Connections (n) | Number of pathways (n) | Predicted SNP value | | |
|---|---|---|---|---|---|---|
| | | | | MY | FY | AFC |
| PPAP2B | phosphatidic acid phosphatase type 2B | 8 | 1 | -0.0689 | -0.0029 | 0.0003 |
| CREBBP | CREB binding protein | 8 | 1 | -0.0574 | -0.0029 | 0.0002 |
| BTRC | beta-transducin repeat containing E3 ubiquitin protein ligase | 8 | 1 | 0.0313 | 0.0008 | -0.0005 |
| ITGAL | integrin subunit alpha L | 8 | 1 | -0.1526 | -0.0051 | 0.0003 |
| LCP2 | lymphocyte cytosolic protein 2\|tyrosine phosphoprotein slp-76 | 8 | 1 | 0.1013 | -0.0010 | 0.0007 |
| CACNA2D1 | calcium voltage-gated channel auxiliary subunit alpha2delta 1 | 7 | 2 | 0.1799 | 0.0065 | 0.0004 |
| LAMA5 | laminin subunit alpha 5 | 7 | 1 | -0.1147 | -0.0035 | 0.0005 |
| CACNA1E | calcium voltage-gated channel subunit alpha1 E | 7 | 2 | 0.0402 | -0.0007 | -0.0001 |
| COL9A3 | collagen type IX alpha 3 chain | 7 | 1 | 0.1454 | 0.0040 | -0.0008 |
| COL2A1 | collagen type II alpha 1 chain | 7 | 1 | -0.1334 | -0.0028 | -0.0003 |
| GABBR2 | gamma-aminobutyric acid type B receptor subunit 2 | 7 | 1 | -0.5772 | -0.0079 | 0.0001 |
| ITPR2 | inositol 1,4,5-triphosphate receptor, type 2 | 7 | 7 | -0.1070 | -0.0069 | -0.0004 |
| HTR4 | 5-hydroxytryptamine receptor 4 | 7 | 1 | 0.2680 | 0.0092 | -0.0008 |
| COL4A5 | collagen type IV alpha 5 chain | 7 | 1 | 0.2224 | 0.0044 | 0.0002 |
| ITPR1 | IP3 receptor | 7 | 8 | 0.0568 | -0.0005 | -0.0001 |
| PTK2B | protein tyrosine kinase 2 beta | 7 | 2 | -0.0314 | 0.0020 | -0.0004 |
| LAMC2 | laminin subunit gamma 2 | 7 | 1 | -0.1495 | -0.0040 | -0.0004 |
| AGPAT4 | lysophosphatidic acid acyltransferase delta | 7 | 1 | 0.1965 | 0.0089 | 0.0004 |
| LAMA3 | laminin subunit alpha 3 | 7 | 1 | -0.1197 | -0.0020 | -0.0005 |
| MAP2K6 | mitogen-activated protein kinase kinase 6 | 7 | 2 | -0.0432 | -0.0053 | 0.0008 |
| WNT11 | Wnt family member 11 | 7 | 1 | -0.0676 | -0.0029 | 0.0004 |
| CHUK | conserved helix-loop-helix ubiquitous kinase | 7 | 2 | -0.0794 | -0.0052 | 0.0007 |
| ERBB4 | erb-b2 receptor tyrosine kinase 4 | 7 | 1 | -0.4089 | -0.0075 | 0.0008 |
| PPP3CC | protein phosphatase 3 catalytic subunit gamma | 7 | 7 | 0.2263 | 0.0028 | -0.0005 |

| Gene name | Gene description | Number of Connections (n) | Number of pathways (n) | Predicted SNP value | | |
|---|---|---|---|---|---|---|
| | | | | MY | FY | AFC |
| PLCL1 | phospholipase C like 1 | 6 | 1 | -0.1320 | -0.0029 | -0.0004 |
| RYR2 | ryanodine receptor 2 | 6 | 4 | -0.5329 | -0.0123 | 0.0009 |
| GABRB1 | gamma-aminobutyric acid type A receptor beta1 subunit | 6 | 1 | -0.1689 | -0.0016 | -0.0001 |
| PTGER3 | prostaglandin E receptor 3 | 6 | 1 | 0.0961 | 0.0031 | -0.0004 |
| TIAM1 | T-cell lymphoma invasion and metastasis 1 | 6 | 2 | 0.0936 | 0.0018 | -0.0009 |
| COL4A2 | collagen type IV alpha 2 chain | 6 | 1 | -0.0612 | -0.0014 | 0.0009 |
| COL4A1 | collagen type IV alpha 1 chain | 6 | 1 | -0.3017 | -0.0021 | -0.0005 |
| WNT10B | Wnt family member 10B | 6 | 1 | -0.1215 | -0.0028 | 0.0004 |
| ABL1 | ABL proto-oncogene 1, non-receptor tyrosine kinase | 6 | 1 | 0.0337 | 0.0005 | -0.0005 |
| WIF1 | WNT inhibitory factor 1 | 6 | 1 | 0.0732 | 0.0030 | -0.0007 |
| PIP5K1B | phosphatidylinositol-4-phosphate 5-kinase type 1 beta | 6 | 1 | -0.2155 | -0.0055 | 0.0012 |
| WNT9B | Wnt family member 9B | 6 | 1 | 0.1068 | 0.0018 | -0.0005 |
| LEF1 | lymphoid enhancer binding factor 1 | 6 | 1 | 0.2246 | 0.0030 | 0.0004 |
| TAB2 | TGF-beta activated kinase 1/MAP3K7 binding protein 2 | 6 | 1 | -0.2206 | -0.0054 | 0.0009 |
| MAP2K4 | mitogen-activated protein kinase kinase 4 | 6 | 1 | 0.0579 | 0.0063 | -0.0004 |
| APC | APC, WNT signaling pathway regulator | 6 | 1 | -0.1126 | -0.0056 | 0.0002 |
| TSC2 | tuberous sclerosis 2 | 6 | 1 | -0.1447 | -0.0031 | 0.0004 |
| CHAD | chondroadherin | 5 | 1 | -0.0792 | -0.0021 | 0.0003 |
| CD38 | CD38 molecule | 5 | 3 | 0.0133 | 0.0002 | -0.0002 |
| DGKH | diacylglycerol kinase eta | 5 | 1 | -0.3457 | -0.0089 | 0.0005 |
| LAMA4 | laminin subunit alpha 4 | 5 | 1 | 0.0531 | 0.0022 | 0.0000 |
| TNR | tenascin R | 5 | 1 | -0.2861 | -0.0101 | 0.0008 |
| GABRA5 | gamma-aminobutyric acid type A receptor alpha5 subunit | 5 | 1 | 0.3057 | 0.0073 | 0.0008 |

| Gene name | Gene description | Number of Connections (n) | Number of pathways (n) | Predicted SNP value | | |
|---|---|---|---|---|---|---|
| | | | | MY | FY | AFC |
| GABRB2 | gamma-aminobutyric acid type A receptor beta2 subunit | 5 | 1 | -0.0758 | -0.0055 | 0.0007 |
| DGKE | diacylglycerol kinase epsilon | 5 | 1 | 0.1128 | 0.0027 | -0.0004 |
| THBS2 | thrombospondin 2 | 5 | 1 | 0.1985 | 0.0036 | -0.0003 |
| PARVA | parvin alpha | 5 | 1 | 0.0972 | 0.0020 | -0.0012 |
| DGKG | diacylglycerol kinase gamma | 5 | 1 | 0.1202 | 0.0024 | -0.0004 |
| DGKB | diacylglycerol kinase beta | 5 | 1 | 0.1431 | 0.0091 | -0.0003 |
| DGKI | diacylglycerol kinase iota | 5 | 1 | -0.3973 | -0.0149 | 0.0015 |
| SGMS2 | sphingomyelin synthase 2 | 5 | 1 | -0.0663 | -0.0022 | 0.0005 |
| GPHN | gephyrin | 5 | 1 | -0.2153 | -0.0089 | 0.0005 |
| SGMS1 | sphingomyelin synthase 1 | 5 | 1 | -0.1802 | 0.0002 | 0.0001 |
| CERS6 | ceramide synthase 6 | 5 | 1 | 0.1091 | 0.0027 | 0.0007 |
| DEGS1 | delta 4-desaturase, sphingolipid 1 | 5 | 1 | -0.0989 | -0.0035 | 0.0008 |
| CERS3 | ceramide synthase 3 | 5 | 1 | 0.0488 | 0.0055 | 0.0004 |
| TNC | tenascin C | 5 | 1 | -0.1908 | -0.0050 | 0.0009 |
| ARRB1 | arrestin beta 1 | 5 | 1 | -0.2640 | -0.0096 | 0.0010 |
| TCF7L2 | transcription factor 7 like 2 | 5 | 1 | 0.3306 | 0.0073 | -0.0007 |
| PPP3R1 | protein phosphatase 3 regulatory subunit B, alpha | 5 | 6 | 0.1239 | 0.0036 | -0.0003 |
| KIF5A | kinesin family member 5A | 4 | 1 | 0.1263 | 0.0062 | -0.0014 |
| KIF5C | kinesin family member 5C | 4 | 1 | 0.1169 | 0.0026 | 0.0003 |
| MAP2K5 | mitogen-activated protein kinase kinase 5 | 4 | 3 | 0.0041 | 0.0036 | 0.0008 |
| MAGI3 | membrane associated guanylate kinase, WW and PDZ domain containing 3 | 4 | 1 | 0.1537 | 0.0036 | -0.0003 |
| RAPGEF6 | Rap guanine nucleotide exchange factor 6 | 4 | 1 | 0.1269 | 0.0062 | 0.0001 |
| RAPGEF2 | Rap guanine nucleotide exchange factor 2 | 4 | 2 | -0.1135 | -0.0018 | -0.0003 |
| GRIK3 | Glutamate Ionotropic Receptor Kainate Type Subunit 3 | 4 | 1 | -0.1952 | -0.0061 | 0.0007 |

| Gene name | Gene description | Number of Connections (n) | Number of pathways (n) | Predicted SNP value | | |
|---|---|---|---|---|---|---|
| | | | | MY | FY | AFC |
| LPAR1 | lysophosphatidic acid receptor 1 | 4 | 3 | 0.3706 | 0.0044 | 0.0000 |
| NTRK2 | neurotrophic receptor tyrosine kinase 2 | 4 | 1 | -0.0573 | -0.0025 | -0.0003 |
| FBXW11 | F-box and WD repeat domain containing 11 | 4 | 1 | -0.1434 | -0.0017 | 0.0012 |
| PLA2G16 | phospholipase A2, group XVI | 4 | 1 | -0.0862 | -0.0029 | 0.0003 |
| GABRG2 | Gamma-Aminobutyric Acid Type A Receptor Gamma2 Subunit | 4 | 1 | -0.0310 | 0.0022 | 0.0005 |
| NFATC2 | nuclear factor of activated T-cells 2 | 4 | 3 | 0.2549 | 0.0052 | -0.0002 |
| RPS6KA2 | ribosomal protein S6 kinase A2 | 4 | 1 | -0.1595 | -0.0050 | 0.0009 |
| RPS6KA3 | ribosomal protein S6 kinase A3 | 4 | 1 | 0.1632 | 0.0025 | -0.0005 |
| MAPKAPK 2 | mitogen-activated protein kinase-activated protein kinase 2 | 4 | 1 | 0.1493 | -0.0005 | -0.0008 |
| MRVI1 | murine retrovirus integration site 1 homolog | 4 | 1 | 0.1175 | 0.0071 | -0.0017 |
| SLC8A3 | solute carrier family 8 member A3 | 3 | 2 | 0.1401 | -0.0004 | 0.0000 |
| SLC8A1 | solute carrier family 8 member A1 | 3 | 2 | 0.1290 | -0.0007 | 0.0011 |
| PRKD1 | protein kinase D1 | 3 | 1 | 0.0053 | 0.0001 | 0.0005 |
| MAGI1 | membrane associated guanylate kinase, WW and PDZ domain containing 1 | 3 | 1 | -0.1226 | -0.0041 | 0.0007 |
| STK3 | serine/threonine kinase 3 | 3 | 1 | 0.0143 | -0.0045 | 0.0000 |
| DKK4 | dickkopf WNT signaling pathway inhibitor 4 | 3 | 1 | 0.1295 | 0.0028 | -0.0006 |
| BID | BH3 Interacting Domain Death Agonist | 3 | 1 | 0.0682 | 0.0031 | -0.0004 |
| CAV2 | caveolin 2 | 3 | 1 | 0.1084 | 0.0025 | -0.0003 |
| ADRA1A | adrenoceptor alpha 1A | 3 | 2 | 0.1735 | 0.0031 | -0.0008 |
| SGPP1 | sphingosine-1-phosphate phosphatase 1 | 3 | 1 | -0.0695 | -0.0038 | 0.0004 |
| ITPKB | inositol-trisphosphate 3-kinase B | 3 | 1 | 0.0341 | 0.0009 | -0.0002 |
| PLA2G2F | phospholipase A2 group IIF | 3 | 2 | -0.1150 | -0.0027 | 0.0005 |
| GABRA3 | gamma-aminobutyric acid type A receptor alpha3 subunit | 3 | 1 | -0.0760 | -0.0027 | 0.0004 |
| FRAT2 | FRAT2, WNT signaling pathway regulator | 3 | 1 | -0.0787 | -0.0023 | 0.0003 |

| Gene name | Gene description | Number of Connections (n) | Number of pathways (n) | Predicted SNP value | | |
|---|---|---|---|---|---|---|
| | | | | MY | FY | AFC |
| SKAP1 | src kinase associated phosphoprotein 1 | 3 | 1 | 0.1007 | 0.0047 | 0.0007 |
| DKK2 | dickkopf WNT signaling pathway inhibitor 2 | 3 | 1 | 0.2370 | 0.0062 | -0.0005 |
| KCNMB2 | potassium calcium-activated channel subfamily M regulatory beta subunit 2 | 3 | 1 | -0.0245 | -0.0024 | 0.0006 |
| KCNMA1 | potassium calcium-activated channel subfamily M alpha 1 | 3 | 2 | -0.1482 | -0.0047 | 0.0004 |
| PRKCE | protein kinase C epsilon | 3 | 2 | 0.6961 | 0.0250 | 0.0001 |
| PLA2G12B | phospholipase A2 group XIIB | 3 | 2 | 0.0602 | 0.0020 | -0.0008 |
| PTPRR | protein tyrosine phosphatase, receptor type R | 3 | 1 | 0.0227 | 0.0035 | 0.0006 |
| RPS6KA5 | ribosomal protein S6 kinase A5 | 3 | 2 | -0.0029 | -0.0017 | -0.0002 |
| DUSP10 | dual specificity phosphatase 10 | 3 | 1 | 0.0818 | 0.0023 | -0.0004 |
| NLK | nemo like kinase | 3 | 2 | 0.0044 | 0.0010 | 0.0000 |
| FYB | FYN binding protein | 3 | 1 | 0.1952 | 0.0020 | -0.0011 |
| MKNK1 | MAP Kinase Interacting Serine/Threonine Kinase 1 | 2 | 1 | -0.1395 | -0.0081 | 0.0006 |
| ETS1 | ETS proto-oncogene 1, transcription factor | 2 | 1 | -0.1685 | -0.0060 | 0.0004 |
| ATP2B2 | ATPase plasma membrane Ca2+ transporting 2 | 2 | 3 | -0.0144 | 0.0010 | -0.0007 |
| ATP2B4 | ATPase plasma membrane Ca2+ transporting 4 | 2 | 3 | 0.0709 | 0.0043 | -0.0010 |
| DNM2 | dynamin 2 | 2 | 1 | -0.1009 | -0.0027 | 0.0005 |
| STK4 | serine/threonine kinase 4 | 2 | 2 | 0.2030 | 0.0048 | 0.0006 |
| RBX1 | Ring-Box 1 | 2 | 1 | -0.1248 | -0.0023 | -0.0003 |
| PPP1R12A | protein phosphatase 1 regulatory subunit 12A | 2 | 3 | -0.1472 | -0.0028 | -0.0003 |
| PIK3R6 | phosphoinositide-3-kinase regulatory subunit 6 | 2 | 3 | -0.0859 | -0.0026 | -0.0003 |
| CTSD | cathepsin D | 2 | 1 | -0.0841 | -0.0019 | 0.0003 |
| TRPC6 | transient receptor potential cation channel subfamily C member 6 | 2 | 1 | 0.1704 | 0.0066 | -0.0007 |
| LOC529488 | glutamate decarboxylase 1 | 2 | 1 | -0.0682 | -0.0010 | 0.0002 |
| GLS | glutaminase | 2 | 2 | 0.1185 | 0.0035 | 0.0003 |

| Gene name | Gene description | Number of Connections (n) | Number of pathways (n) | Predicted SNP value | | |
|---|---|---|---|---|---|---|
| | | | | MY | FY | AFC |
| GAD2 | glutamate decarboxylase 2 | 2 | 1 | 0.0231 | 0.0002 | 0.0003 |
| FCER1A | Fc fragment of IgE receptor Ia | 2 | 2 | -0.1174 | -0.0050 | 0.0005 |
| MS4A2 | Membrane Spanning 4-Domains A2 | 2 | 2 | -0.1083 | -0.0027 | 0.0004 |
| ARF1 | ADP ribosylation factor 1 | 2 | 1 | 0.1142 | 0.0021 | 0.0004 |
| SHANK2 | SH3 and multiple ankyrin repeat domains 2 | 2 | 1 | 0.2879 | 0.0045 | -0.0019 |
| DLGAP1 | DLG associated protein 1 | 2 | 1 | -0.1384 | -0.0029 | 0.0008 |
| SHANK1 | SH3 and multiple ankyrin repeat domains 1 | 2 | 1 | 0.1503 | 0.0044 | -0.0003 |
| TCF7L1 | transcription factor 7 like 1 | 2 | 1 | 0.2505 | 0.0056 | -0.0003 |
| ADRBK2 | G Protein-Coupled Receptor Kinase 2 | 2 | 1 | -0.0983 | -0.0019 | -0.0011 |
| PPM1B | protein phosphatase, Mg2+/Mn2+ dependent 1B | 1 | 1 | 0.0792 | 0.0019 | -0.0004 |
| CFTR | ATP-binding cassette sub-family C member 7 | 1 | 1 | 0.2142 | 0.0067 | -0.0008 |
| DNM3 | dynamin 3 | 1 | 1 | 0.2789 | 0.0047 | 0.0006 |
| ARHGAP35 | Rho GTPase activating protein 35 | 1 | 1 | 0.0212 | 0.0008 | 0.0000 |
| IL1R1 | interleukin 1 receptor type 1 | 1 | 1 | 0.4206 | 0.0096 | 0.0003 |
| SLC17A8 | solute carrier family 17 member 8 | 1 | 1 | -0.0982 | -0.0034 | 0.0006 |
| SLC18A2 | solute carrier family 18 member A2 | 1 | 1 | -0.0805 | -0.0023 | -0.0006 |
| MECOM | MDS1 and EVI1 complex locus | 1 | 1 | -0.4502 | -0.0107 | -0.0001 |
| KSR2 | kinase suppressor of ras 2 | 1 | 1 | -0.1042 | -0.0044 | -0.0001 |
| MYLK3 | myosin light chain kinase 3 | 1 | 4 | -0.0749 | -0.0022 | 0.0003 |
| PAK7 | P21 (RAC1) Activated Kinase 5 | 1 | 2 | 0.2659 | 0.0046 | 0.0002 |
| PARD3 | par-3 family cell polarity regulator | 1 | 1 | 0.1968 | 0.0051 | -0.0002 |
| DOCK4 | dedicator of cytokinesis 4 | 1 | 1 | 0.1897 | 0.0068 | 0.0007 |
| DOCK1 | dedicator of cytokinesis 1 | 1 | 1 | -0.3004 | -0.0127 | -0.0011 |
| TGFB2 | transforming growth factor beta 2 | 1 | 1 | 0.1360 | 0.0038 | -0.0011 |
| MYL12B | myosin, light chain 12B, regulatory | 1 | 1 | -0.1608 | -0.0021 | 0.0010 |
| P2RX1 | purinergic receptor P2X 1 | 1 | 1 | -0.0748 | -0.0020 | 0.0005 |

| Gene name | Gene description | Number of Connections (n) | Number of pathways (n) | Predicted SNP value | | |
|---|---|---|---|---|---|---|
| | | | | **MY** | **FY** | **AFC** |
| P2RX3 | purinergic receptor P2X 3 | 1 | 1 | -0.0956 | 0.0019 | 0.0003 |
| SIPA1L1 | signal induced proliferation associated 1 like 1 | 1 | 1 | 0.0693 | 0.0043 | 0.0005 |
| GRIK4 | glutamate ionotropic receptor kainate type subunit 4 | 1 | 1 | -0.3617 | -0.0093 | 0.0008 |
| MAPT | PHF-tau\|microtubule associated protein tau\|neurofibrillary tangle protein\|paired helical filament-tau | 1 | 1 | 0.0848 | 0.0019 | -0.0003 |
| PHKB | phosphorylase kinase regulatory subunit beta | 1 | 1 | -0.1028 | -0.0029 | 0.0002 |
| PHKA2 | Phosphorylase Kinase Regulatory Subunit Alpha 2 | 1 | 1 | 0.1117 | 0.0032 | -0.0003 |
| RAP1GAP | RAP1 GTPase activating protein | 1 | 1 | 0.0637 | 0.0002 | 0.0003 |
| **Total** | | **2,313** | **625** | **-5.6150** | **-0.1404** | **0.0067** |
